# Spatially explicit accuracy assessment of deep learning-based, fine-resolution built-up land data in the United States

**DOI:** 10.1016/j.jag.2023.103469

**Published:** 2023-08-28

**Authors:** Johannes H. Uhl, Stefan Leyk

**Affiliations:** a University of Colorado Boulder, Institute of Behavioral Science, 483 UCB, Boulder, CO 80309, USA; b University of Colorado Boulder, Cooperative Institute for Research in Environmental Sciences (CIRES), 216 UCB, Boulder, CO 80309, USA; c University of Colorado Boulder, Department of Geography, 260 UCB, Boulder, CO 80309, USA

**Keywords:** Global human settlement layer, Sentinel-2, Spatially explicit accuracy assessment, Explainable AI, Deep learning, Built-up areas

## Abstract

Geospatial datasets derived from remote sensing data by means of machine learning methods are often based on probabilistic outputs of abstract nature, which are difficult to translate into interpretable measures. For example, the Global Human Settlement Layer GHS-BUILT-S2 product reports the probability of the presence of built-up areas in 2018 in a global 10 m × 10 m grid. However, practitioners typically require interpretable measures such as binary surfaces indicating the presence or absence of built-up areas or estimates of sub-pixel built-up surface fractions. Herein, we assess the relationship between the built-up probability in GHS-BUILT-S2 and reference built-up surface fractions derived from a highly reliable reference database for several regions in the United States. Furthermore, we identify a binarization threshold using an agreement maximization method that creates binary built-up land data from these built-up probabilities. These binary surfaces are input to a spatially explicit, scale-sensitive accuracy assessment which includes the use of a novel, visual-analytical tool which we call focal precision-recall signature plots. Our analysis reveals that a threshold of 0.5 applied to GHS-BUILT-S2 maximizes the agreement with binarized built-up land data derived from the reference built-up area fraction. We find high levels of accuracy (i.e., county-level F-1 scores of almost 0.8 on average) in the derived built-up areas, and consistently high accuracy along the rural–urban gradient in our study area. These results reveal considerable accuracy improvements in human settlement models based on Sentinel-2 data and deep learning, as compared to earlier, Landsat-based versions of the Global Human Settlement Layer.

## 1. Introduction

Accurately mapping the spatial distribution and dynamics of human settlements at planetary scale is critical for monitoring and understanding global processes such as (sub)urbanization, land take, rural–urban transformations, the dynamics of the wildland-urban interface and other human-nature coupled systems. To understand, mitigate, or adapt to pressing issues related to these processes, such as biodiversity loss, overpopulation or increasing social inequality, and to ensure sustainable urban and rural development, stakeholders, planners, and researchers often use remote sensing-based land use, land cover, or settlement data as a basis for decision making. For example, the change rate of built-up area over time with respect to population change is an important indicator for sustainable urban development (Corbane et al., 2018, Ehrlich et al., 2018, Melchiorri et al., 2019, Cai et al., 2020) which may, alongside other demographic or socio-economic metrics, drive political decisions at a local scale but also at a country level or even in a global context. Specifically, the Sustainable Development Goal (SDG) indicator 11.3.1. quantifies the land use efficiency of a city, which is directly related to the level of sustainability: While compact cities provide access to public services at lower cost, sprawling cities (i.e., more built-up surface increase per population increase) typically require increased demand for mobility (e.g., commuting effort) and thus, increased energy consumption. (UN Habitat; 2018).

Thus, the accuracy of the data underlying such indicators and decisions is critical. Global, high-resolution, and typically multitemporal datasets on built-up areas have emerged in recent years, catalyzed by the availability of long-term remote sensing archives (e.g., Landsat), the more recently launched Sentinel-1 and Sentinel-2 platforms, and by technological advances facilitating data access and processing, such as Google Earth Engine (Gorelick et al., 2017) or Deep Learning (Ball et al., 2017, Zhu et al., 2017). Such datasets include the Global Human Settlement Layer (GHSL; Pesaresi et al., 2013), Global Artificial Impervious Area (GAIA; Gong et al., 2020), Global Urban Footprint (Esch et al., 2017) and its successor, the World Settlement Footprint (WSF; Marconcini et al., 2020a) including the multi-temporal dataset WSF-Evolution (Marconcini et al., 2020b), as well as the High Resolution Settlement Layer (HRSL; Facebook Connectivity Lab & CIESIN 2016). Moreover, industry-driven efforts have sparked the availability of building footprint and road network data at a continental or nearly-planetary scale^1,2,3^ (Sirko et al., 2021), complemented by Volunteered Geographic Information (i.e., OpenStreetMap, OSM). Such community-based, participatory mapping efforts can be useful for local, timely data acquisition, for example in the case of disaster response (Herfort et al., 2021). While these datasets represent considerable improvements regarding their spatial resolution as compared to older data products (e. g., Defourny et al., 2006, Balk et al., 2006), there is an urgent demand for quantifying the accuracy of these datasets, to enable informed, reflected, and uncertainty-aware data interpretation and decision making. This is particularly important as such datasets are commonly used for population disaggregation (Freire et al., 2015, Leyk et al., 2019, Palacios-Lopez et al., 2019).

The GHS-BUILT-S2, which is tested herein, is one of the input layers for the most recent generation of the GHS-BUILT data suite (Schiavina et al., 2022), Thus, quantitative knowledge of its accuracy is crucial for the evaluation of the follow-up data products mapping different components of the built environment, rural–urban classes, as well as population distributions. Herein, we use our reference data employed in earlier studies, and conduct a spatially explicit accuracy assessment of the GHS-BUILT-S2 dataset, building up on our previous work (Section 2). The GHS-BUILT-S2 dataset is particularly interesting, as it reports the probability of being built-up for each grid cell, whereas previous GHS-BUILT versions provided binary built-up / not built-up labels. These “built-up probabilities” are the output of a deep learning-based classifier, and indicate the presence of built-up surface. Built-up land data is commonly used for (a) population disaggregation, (b) quantification of built-up surface, density, and its variations within an area of interest, or (c) Characterization of the morphology of urban spaces or the built environment. All these applications require either Boolean (built-up / not built-up) data, or a continuous measure of built-up surface. Thus, the translation of these probability values to a meaningful measure of built-up area is crucial for the unbiased usability of the GHS-BUILT-S2. This problem, in a general sense, is a common issue in the broader context of explainable and interpretable artificial intelligence (Gunning et al., 2019, Papadakis et al., 2022), and contributes to a growing body of literature focusing on uncertainty-aware applications of deep learning in the field of remote sensing (Maxwell et al., 2021a,b). Thus, in this work, we pose the following questions:

1. How can built-up probabilities be translated into a meaningful, physical measure of built-up area?
2. How accurate is the GHS-BUILT-S2 across regions in the United States, and across the rural–urban continuum, in a localized context?
3. How sensitive are the obtained, spatially explicit, thematic accuracy estimates to grid misalignment and to the chosen spatial unit?

We try to answer these questions by 1) analyzing the relationship between built-up probability and built-up area fraction, and performing an agreement analysis to find the “optimal” threshold for converting built-up probabilities in GHS-BUILT-S2 to binary layers of built-up land, 2) conducting a rigorous, spatially explicit accuracy assessment against our reference data, and 3) assessing the sensitivity of our accuracy estimates to various parameters used in the accuracy assessment. For the spatially explicit accuracy assessment conducted in step 2) we present a novel visual-analytical method to assess local accuracy variations within a given (focal) region, which we call “focal precision-recall signature plots”. In the following, we provide an overview of related work (Section 2), describe our data and methods (Section 3), present and discuss our results (Section 4), and conclude with some final remarks (Section 5).

## 2. Related work and contributions of this study

To meet the demand for quantitative knowledge on the uncertainty in human settlement datasets, researchers have carried out data integration and evaluation efforts to facilitate accuracy assessments of the GHSL built-up area layers at 38 m or 30 m resolution (Blei et al., 2018, Leyk et al., 2018, Liu et al., 2020), of OSM (Hecht et al., 2013, Fan et al., 2014, Brovelli & Zamboni 2018), of WSF (Marconcini et al., 2020a), or cross-comparisons of several of the aforementioned datasets (Klotz et al., 2016). Such evaluation efforts also include studies focusing on specific regions (Mück et al., 2017, Sliuzas et al., 2017, Leyk et al., 2018, Liu et al., 2020, Tripathy & Balakrishnan 2021), geographic concepts (e.g., the rural–urban continuum; Leyk et al., 2018, Uhl et al., 2018, Uhl & Leyk 2022a, Uhl & Leyk 2022b), rural areas in particular (Kaim et al., 2022, Wang et al., 2022), or different landscape and settlement types (e. g., informal settlements; Van Den Hoek and Friedrich, 2021). Related to data quality issues, the implications of discrepancies between population-based and built-up area based urban definitions have been studied (Balk et al., 2018, Leyk et al., 2019).

However, few studies have explored the accuracy of built-up surface datasets over time. This is due to a lack of multi-temporal, independently compiled reference data of presumably higher accuracy than the data under test (FGDC 1998). Thus, in previous work, we used an integrated dataset of cadastral parcel data and building footprint data, including construction year information, to generate multi-temporal ground truth data of built-up surface at arbitrary points in time. This work was carried out in 2016, when no open country-wide building footprint dataset or open harmonized parcel dataset existed. Specifically, we collected parcel data including construction year information, and building footprint data from cadastral offices for a selection of 33 counties in the United States, where such data were available. We integrated the parcel and building footprint data via spatial joins to create a set of over 6 million building footprints attributed with their construction year (Uhl and Leyk, 2022c). This dataset is the multitemporal building footprint dataset for 33 U.S. counties (MTBF-33) and represents a reliable data source to create snapshots of built-up area for arbitrary points in time between 1900 and 2015. Building construction dates come from public records such as tax assessments, and building footprint data is mostly derived from LiDAR point cloud data or manual digitization.

We employed the MTBF-33 dataset to quantify temporal accuracy trends from 1975 to 2014, in the first version of the Global Human Settlement Layer (GHS-BUILT R2015B; Pesaresi et al., 2016), derived from Landsat data at a resolution of 38 m (Leyk et al., 2018). The same dataset was used to quantify accuracy improvements between the GHS-BUILT R2016A and the GHS-BUILT R2018A (Florczyk et al., 2019), which was available at 30 m resolution, also based on Landsat data, but informed by Sentinel-2 multispectral data available at 10 m resolution, and the GHS-BUILT-S1 (Corbane et al., 2017), derived from Sentinel-1 Synthetic Aperture Radar data at 20 m spatial resolution (Uhl et al., 2018).

While these accuracy assessments were carried out within strata of built-up surface density, approximating the rural–urban continuum, they were based on global accuracy estimates (i.e., calculated per stratum and epoch) possibly neglecting local variability of data accuracy (e. g., Foody 2002, Strahler et al., 2006, Wickham et al., 2018). Hence, we also employed the MTBF-33 dataset to measure local accuracy variations in the GHSL R2018A built-up surfaces using a spatially explicit accuracy assessment, revealing further spatial disentanglements of accuracy trends over time (Uhl & Leyk, 2022a). In that work, we presented an efficient spatial data processing pipeline for the creation of continuous (focal) accuracy surfaces, facilitating spatially explicit agreement assessments of categorical gridded data in general (Uhl & Leyk, 2022a). Moreover, we tested the suitability of commonly used agreement metrics for the quantification of built-up area mapping accuracy, taking into account the varying levels of class imbalance between grid cells labelled as built-up and not built-up along the rural–urban gradient (Uhl & Leyk, 2022a). Moreover, we employed this data processing pipeline to explore the relationship between mapping accuracy and morphological characteristics of the built-up areas (exemplified by the GHS-BUILT R2018A dataset). We employed correlation and regression analyses based on continuous accuracy surfaces, and landscape metrics calculated in the same grid and at the same spatial scales. That study identified strong associations between mapping accuracy and morphology of built-up surfaces, constituting important a priori information for data analysts regarding expected levels of data accuracy (Uhl & Leyk, 2022b).

Other studies demonstrated the usefulness of the MTBF-33 dataset for applications beyond accuracy assessments (e.g., as training data for information extraction in multi-temporal geospatial data; Uhl et al., 2017, Uhl & Leyk, 2020). Therefore, we made the MTBF-33 dataset publicly available (Uhl & Leyk, 2022c). Given the work in Leyk et al. (2018), Uhl et al. (2018), and Uhl & Leyk (2022a) provides a sequence focusing on GHSL data accuracy across different data versions (including different sensors and source data resolutions), at increasing spatial detail, it is a natural next step to apply our evaluation framework to the more recent, GHS-BUILT-S2 dataset available at 10 m spatial resolution.

Thus, the work presented herein will extend the progression of evaluated GHSL versions, in a consistent manner (i.e., using the same study areas covered by the MTBF-33 dataset), documenting potential effects of classifier choice (i.e., Symbolic Machine Learning vs. Convolutional Neural Networks) on mapping accuracy. Moreover, we make several novel methodological contributions: We aim to facilitate the establishment of practical guidelines for data users interested in translating probabilistic classification outputs into meaningful physical measurements and raise awareness of potential spatial variability in these “translation rules”. Finally, in addition to the novel visual-analytical tools for spatially explicit agreement assessments (i.e., focal precision-recall signature plots) presented herein, we conduct a spatially explicit sensitivity analysis aiming to sensibilize data analysts about the effects of positional uncertainty on the outcomes of thematic agreement assessments.

## 3. Data and methods

**Fig. 1.**
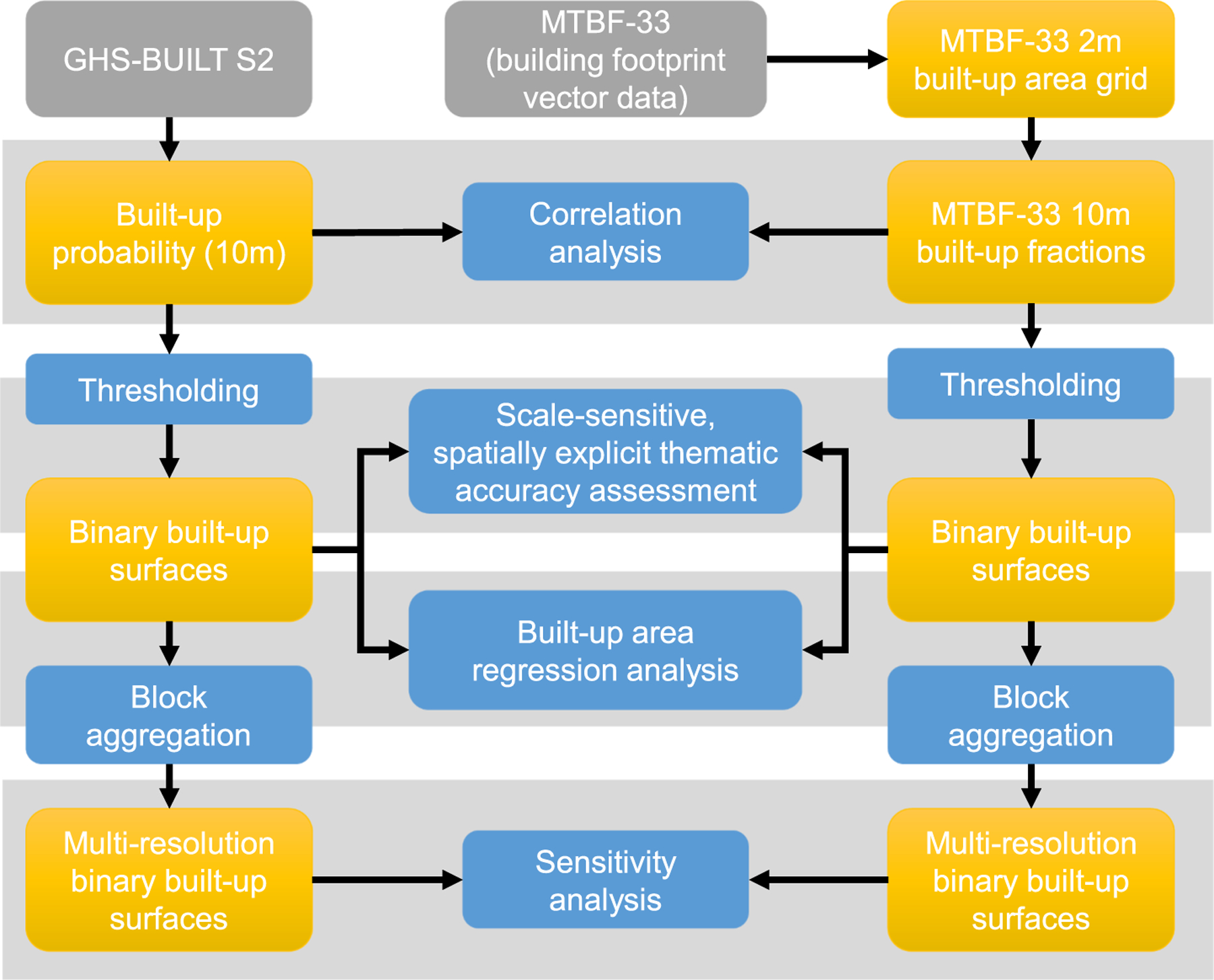
Data processing and analysis flow diagram.

The data used in this study is the GHS-BUILT-S2 dataset, and the MTBF-33 reference dataset (Section 3.1) which are the basis for all analytical steps (Section 3.2). These steps are shown in Fig. 1 and will be described in the following section.

### 3.1. Input data and preprocessing

**Fig. 2.**
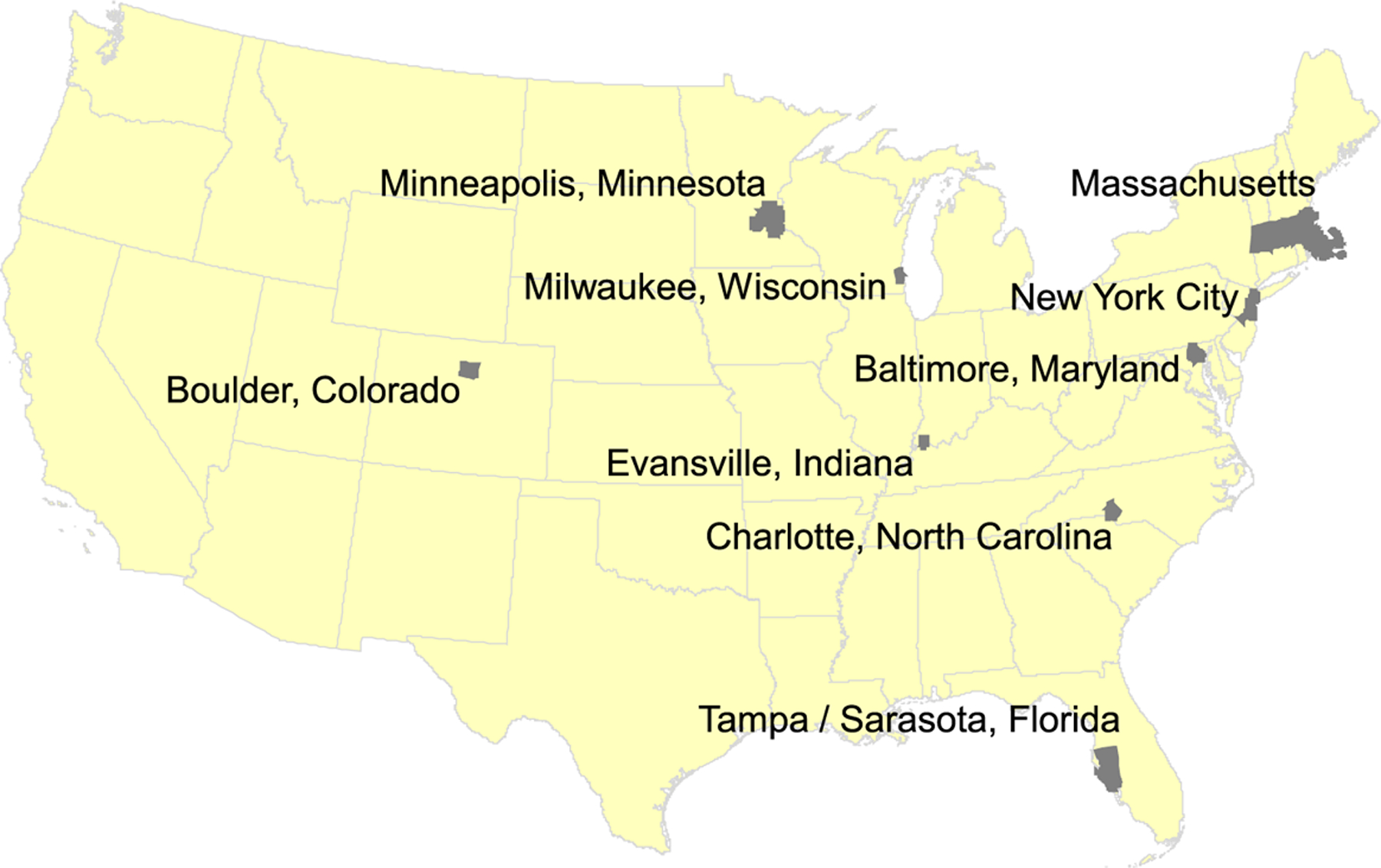
Study area: The 30 counties covered by MTBF-33. These counties can be grouped into nine study regions, which are labelled in the map.

In this work, we used 30 out of the 33 counties covered by the MTBF-33 dataset (Section 2) as study areas (Fig. 2). This is consistent with the study areas in prior work (Leyk et al., 2018, Uhl et al., 2018) and thus, will enable the direct comparison of accuracy measures across different versions of the GHSL, reducing potential effects of uncertainty in the reference data when assessing changes in accuracy across data versions.

**Fig. 3.**
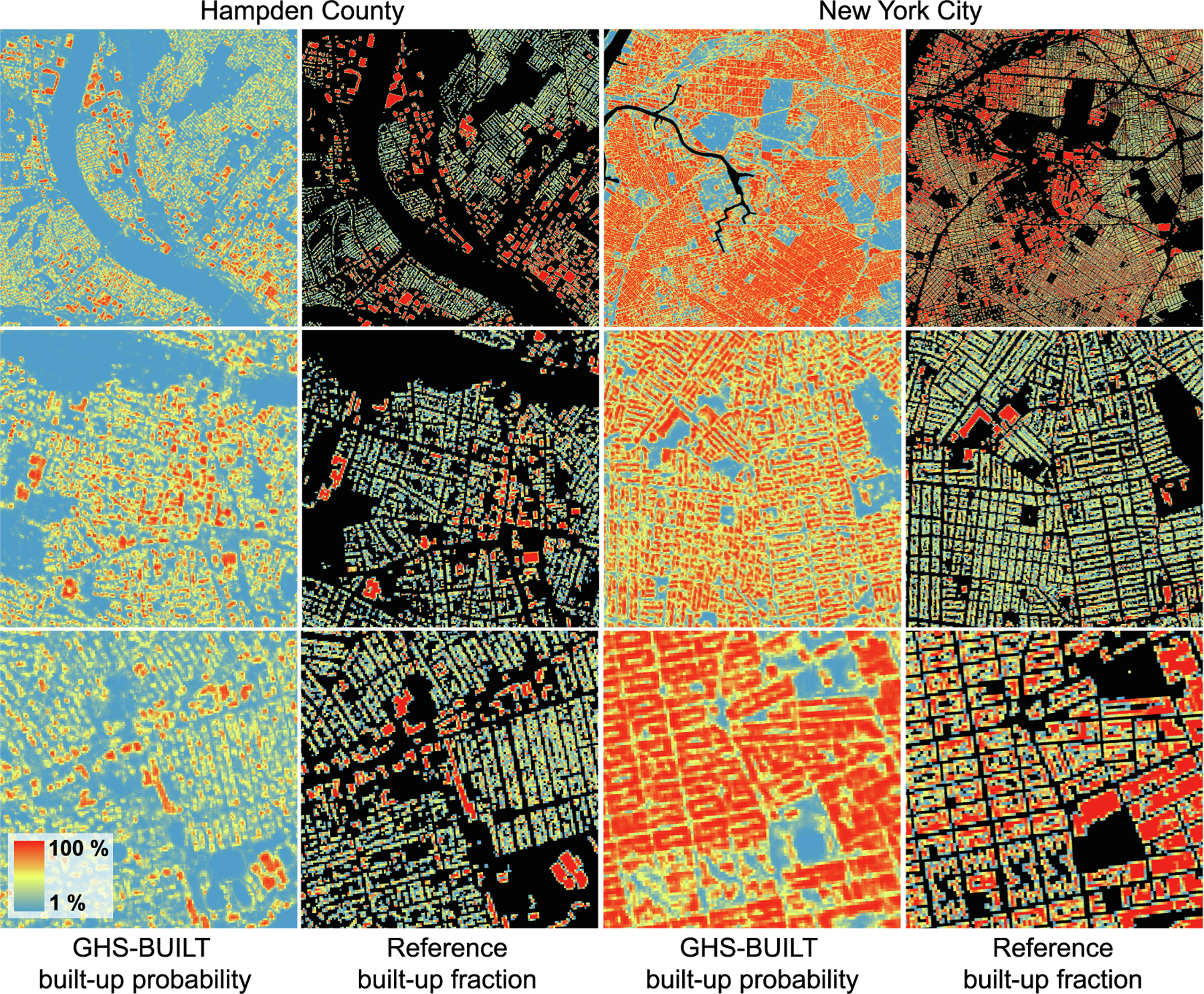
Visual comparison between GHS-BUILT-S2 built-up probabilities and built-up fractions based on the reference data for parts of Hampden County (Massachusetts) and New York City.

**Fig. 4.**
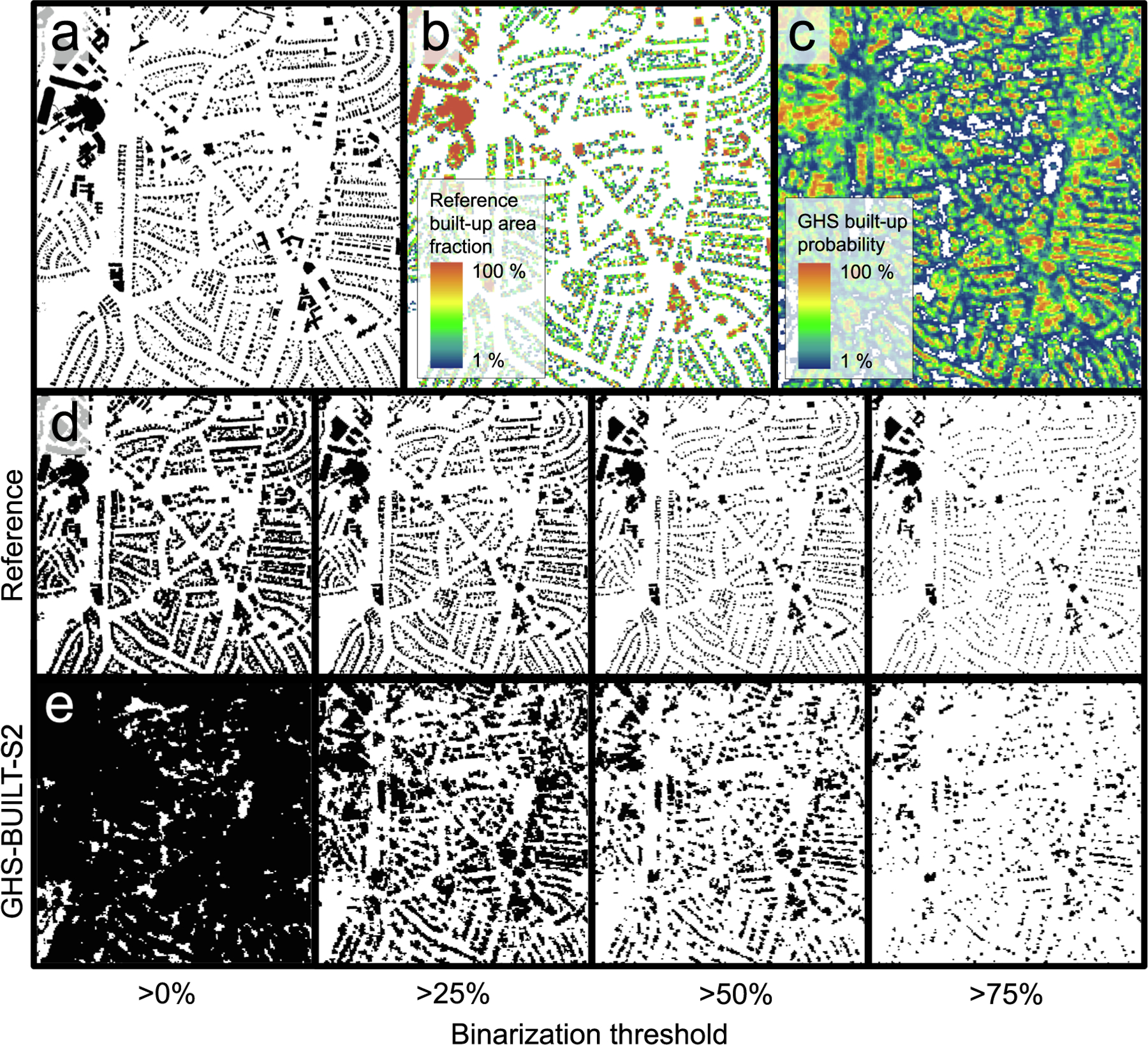
Preprocessing 10 m built-up probabilities and reference data to generate different binary built-up surface layers. (a) Rasterized reference building footprint data at 2 m spatial resolution, derived from building footprint vector data from the MTBF-33 database, (b) reference built-up fraction at 10 m spatial resolution, (c) GHS-BUILT-S2 built-up probability surface at 10 m spatial resolution, (d) reference built-up fractions binarized for a range of thresholds, and (e) the GHS-BUILT-S2 built-up probabilities binarized by the same range of thresholds. All data shown for a part of the city of Charlotte (Mecklenburg County, North Carolina).

For the counties covered by MTBF-33, we obtained the GHS-BUILT-S2 surfaces, available at 10 m spatial resolution, in Mollweide projection (Corbane et al., 2020). Among these counties are Hampden County (Massachusetts) and New York City,^4^ for which we show the GHS-BUILT-S2 in Fig. 3. We then rasterized the MTBF-33 building polygon data to an intermediate spatial resolution of 2 m, aligned to (and nested within) the GHS-BUILT-S2 grid, and then aggregated these 2 m grid cells to the 10 m cells by calculating the proportion of 2 m cells, i.e., the fraction of reference built-up area per grid cell, reported in % (see Fig. 3 for some examples). We did this based on all buildings in MTBF-33 (regardless of their construction date), and thus, approximately representing the building stock in 2015. This rasterization process is also illustrated in Fig. 4 a) and b).

The GHS-BUILT-S2 dataset contains built-up probabilities, which are the output of a convolutional neural network based classifier called *GHS-S2Net* (Corbane et al., 2021). GHS-BUILT-S2 is based on Sentinel-2 multispectral remote sensing data, acquired in 2018. Thus, there is a 3-year temporal offset to our reference data.

### 3.2. Methods

#### 3.2.1. Assessing the relationship between built-up probability and built-up fraction

Based on the grid cells covered by both, GHS-BUILT-S2 and MTBF-33, we visually compared the co-occurrences of built-up probability (henceforth called BUPROB) in GHS-BUILT-S2 and the built-up fraction (henceforth called BUFRAC) derived from the reference data, on a cell-by-cell basis, for each county under study, and across all counties. We also calculated correlations between BUFRAC (see Fig. 4b) and BUPROB (see Fig. 4c) across all counties, and for each county individually. This way we were able to assess whether the relationship between BUPROB and BUFRAC is stationary across regions as an indication of the robustness of the classifier underlying the GHS-BUILT-S2 and its spatial generalization capabilities (Section 4.1). Moreover, we conducted a regression analysis between the two spatial variables, to further test the variability of their relationship across regions. Knowledge of this relationship will be important to better understand the translation of built-up probability into built-up fraction. Specifically, we used regression analysis to test whether BUFRAC can be estimated from BUPROB by fitting a function. We tested a linear model, as well as 2nd, 3rd, and 4th order polynomial functions to allow for a more complex relationship between the two spatial variables, and analyzed regional variability in the model performances.

#### 3.2.2. Generating binary built-up surface layers

For many applications of human settlement data, such as population disaggregation (e.g., Leyk et al., 2019, Palacios-Lopez, 2019) or analyses of urban size and morphology (e.g., Strano et al., 2021, Uhl et al., 2021, Uhl & Leyk, 2022b), practitioners require binary layers of built-up vs. not built-up areas. Thus, a second issue when working with the GHS-BUILT-S2 data product is the thresholding / binarization of the continuous built-up probabilities to create a binary layer. To provide guidelines on how to identify the optimal threshold in the GHS-BUILT-S2 BUPROB values, we used a heuristic approach: We applied four cutoff values (i.e., >0%, >25%, >50%, >75%) to both the reference BUFRAC surfaces (see Fig. 4d), and the BUPROB surfaces (Fig. 4e). We then assessed the thematic agreement between the binarized BUFRAC and BUPROB surfaces, for all 16 threshold combinations, by calculating the F-1 score (van Rijsbergen, 1974) for each combination, for each county, and for sub-county strata that were defined based on reference built-up area density. This is motivated by prior work, that revealed accuracy variations across the rural–urban continuum (Leyk et al., 2018, Uhl et al., 2018, Uhl et al., 2022a, Uhl et al., 2022b; see Section 2) creating the expectation that the relationship between BUPROB and BUFRAC could also vary between rural and urban strata. To do so, we calculated the built-up area density based on the reference built-up area fractions within 1 km × 1 km blocks. In each county, we grouped these blocks into lower-, medium- and higher-density strata, using an equal width classification scheme. Finally, for each county and each of its strata, we identified the threshold combination for which the agreement maximizes (Section 4.2).

#### 3.2.3. Spatially explicit accuracy assessment

**Fig. 5.**
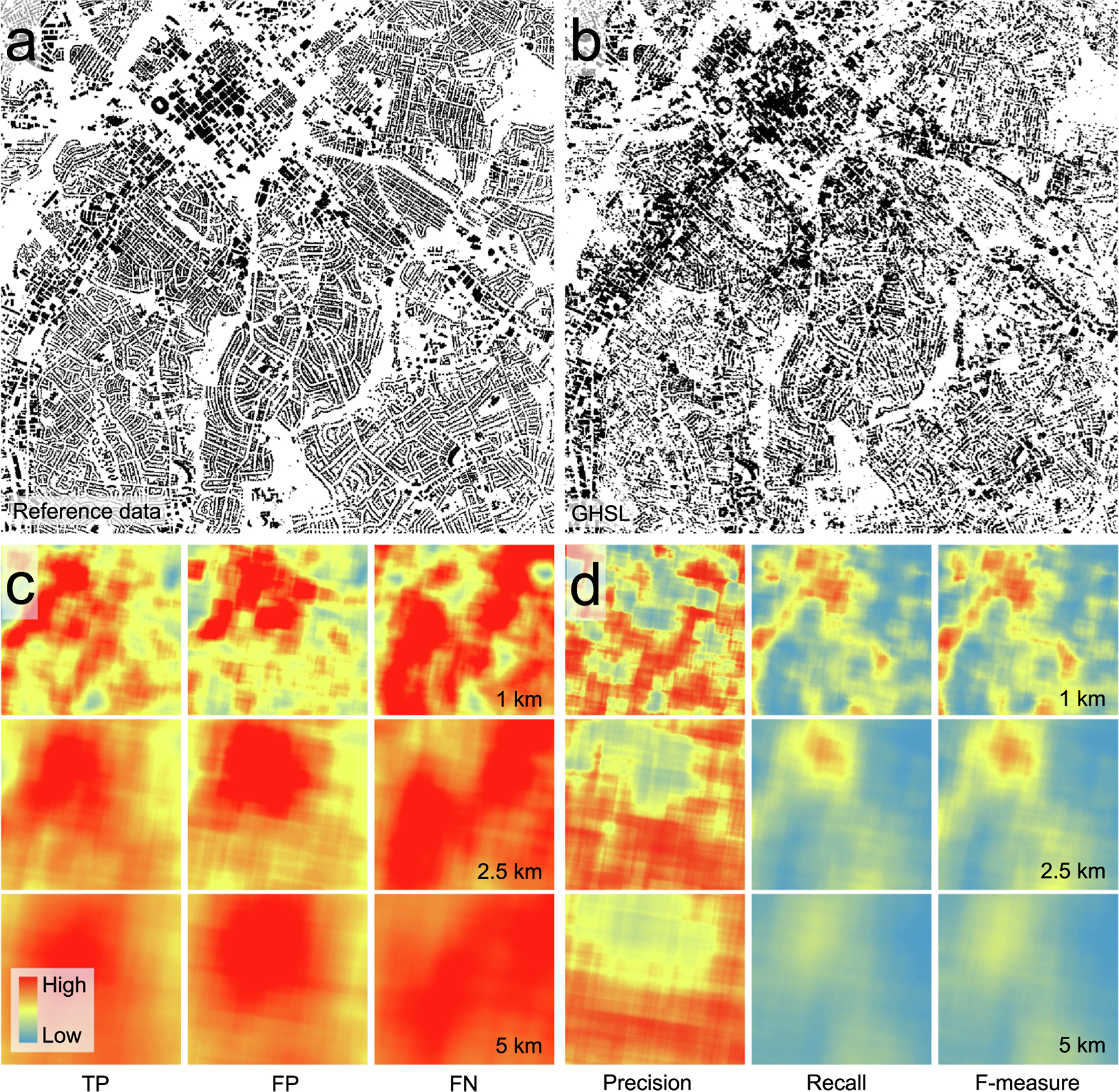
Input and output surfaces of the spatially explicit accuracy assessment. (a) binarized GHS built-up probability (>0.5) and (b) binarized reference built-up fractions (>0), Also shown for different focal window sizes (1 km, 2.5 km and 5 km) are (c) derived localized confusion matrix element surfaces and (d) focal accuracy surfaces. All data shown for a part of the city of Charlotte (Mecklenburg County, North Carolina).

As uncertainty in geospatial data is known to be often spatially non-stationary (Foody, 2002, Leyk and Zimmermann, 2004, Wickham et al., 2018), researchers and analysts increasingly conduct spatially explicit accuracy assessments (Foody 2007, Löw et al., 2013, Khatami et al., 2017, Waldner et al., 2017, Mitchell et al., 2018, Morales-Barquero et al., 2019), rather than reporting overall accuracy metrics which overly generalize the spatial variations of accuracy (Strahler et al., 2006). Hence, once we decided for a cutoff value to binarize both surfaces BUPROB and BUFRAC (based on the method described in Section 3.2.2), we conducted a spatially explicit, thematic accuracy assessment between the two surfaces. To do so, we employed a strategy developed in earlier work (Uhl & Leyk, 2022a; see Section 2), which overlays a binary, gridded test surface (binarized BUPROB) on a reference surface (binarized BUFRAC) (Fig. 5a,b). Specifically, this overlay creates four surfaces, each encoding the presence (1) / absence (0) of the four agreement categories per grid cell (true positives = TP, true negatives = TN, false positives = FP, false negatives = FN). Next, a user-defined kernel convolves over each of these agreement category surfaces and counts the occurrences of TP, FP, and FN within a focal window given by the extent of the kernel. Herein, we used quadratic kernels of size 1 km × 1 km, × 2.5 km × 2.5 km, and 5 km × 5 km, to capture local accuracy at multiple spatial scales. The result of these convolutions are three surfaces per kernel size, holding the counts of TP, FP, and FN grid cells within the focal region around each grid cell, and thus, representing a spatialized (or localized) version of a binary confusion matrix (Fig. 5c).

Note that the TN category is disregarded here, as the true negatives (i.e., areas not built-up in test and reference data) often represent the dominant class, particularly in rural areas, and thus, we do not use agreement metrics involving the count of TN, as they tend to yield inflated or biased values if there is class imbalance (e.g., Rosenfeld & Melley, 1980, Stehman & Wickham, 2020). In a final step, surfaces of focal precision, recall, and F-measure are calculated on a cell-by-cell basis, resulting in spatially exhaustive, spatially explicit agreement metrics between the input surfaces (Fig. 5d), representing focal (or localized) accuracy estimates. These surfaces can then be used for further analyses.

The cell-by-cell sum of focal TP and FN yields the total number of built-up reference grid cells per focal window. Likewise, the cell-by-cell sum of focal TP and FP yields the total number of built-up grid cells in the test data. These counts represent the quantity of built-up area per focal window, which we call “built-up quantity”. By comparing these counts derived from the reference and the test data, we can measure the quantity agreement of built-up area per focal window, while relaxing the constraint of positional alignment, as measured by the cell-by-cell agreement metrics precision, recall, and F-1 score.

Moreover, we use these counts to calculate the reference built-up surface density (henceforth called “built-up density”) per focal window to define density-based strata. We then calculate accuracy metrics within these strata and analyze accuracy trajectories across the rural–urban continuum (i.e., from rural low-density settlements to urban high-density settlements) (Section 4.3).

Herein, we used the created agreeement surfaces to perform the following analyses: We calculated the overall agreement between the binary surfaces 1) per county, 2) within three (equal width) reference built-up density-based strata per county, loosely related to rural, peri*-*urban, and urban areas, and 3) we visually assessed the interactions between localized commission and omission errors, by creating scatterplots of focal precision and focal recall, per county. This is motivated by the findings of previous accuracy assessments, where we found low precision, but high recall in urban areas, as well as low precision and low recall in rural areas, mostly caused by the false detection of roads as built-up areas (e.g., Leyk et al., 2018). Thus, such “signature plots” of focal precision vs focal recall provide a visual-analytical way to assess the overall levels (and distributions) of commission and omission errors, and to detect interactions between them (Section 4.3).

#### 3.2.4. Localized built-up area regression analysis

While we assessed the thematic agreement between the gridded surfaces in Section 3.2.3, we are aware that positional uncertainty in the source datasets may cause misalignment between the gridded reference and test surfaces, which may bias the outcomes of our thematic accuracy assessment as described in Section 3.2.3 (e.g., Congalton, 2007). Hence, we relaxed the constraint of cell-by-cell alignment between test and reference data and used the focal built-up area in both datasets to assess their quantity agreement, disregarding their spatial overlap. We conducted linear regression analyses using the focal built-up quantities in test and reference data, per county, and across all counties, to test the regional variability of the quantity agreement (Section 4.4). This analysis is also motivated by a range of applications of human settlement data that do not require an analytical unit of 10 m grid cells, where coarser spatial resolutions are sufficient (e.g., urban shape analysis using landscape metrics, population disaggregation, etc.).

#### 3.2.5. Sensitivity to positional uncertainty and assessment support

Related to the mentioned potential misalignment between the gridded test and reference surfaces, we analyzed the sensitivity of the agreement measures to potential positional uncertainty in the data. As our reference surfaces are based on cadastral data, there is some positional uncertainty associated with the building footprint polygons, that propagates into our gridded surfaces (Congalton, 2007). Moreover, the Sentinel-2A data underlying the GHS-BUILT-S2 dataset may be affected by positional uncertainty due to image registration and other image processing steps.

To test the sensitivity of our focal accuracy metrics to such potential misalignment, we implemented two sensitivity analyses: 1) For a selection of three counties (i.e., New York City, Hampden County, Massachusetts, and Boulder County, Colorado) we systematically shifted the reference surface by 1 and 2 cells in both x- and y-direction, mimicking offsets between the data of up the 20 m in each direction. We then recalculated the focal accuracy metrics for each combination of shifts, and assessed their variation, on average, as well as spatially explicit (Section 4.6.1).

Moreover, we recalculated our focal accuracy assessments based on aggregated grid cell blocks. For example, if a built-up grid cell at native 10-m resolution does not spatially coincide with a built-up reference cell, but has a built-up reference cell within its 3 × 3 cell neighborhood, the assigned agreement category would still be TP (true positive). Such an aggregation of the analytical unit used for map comparison is a commonly employed technique to relax the requirement of alignment at the native resolution and to account for potential positional discrepancies between datasets which may bias the thematic agreement measures (Congalton, 2007, Gu & Congalton, 2020, Gu & Congalton, 2021, Marconcini et al., 2020a). We did such a block-based accuracy assessment for all counties using 3 × 3 and 5 × 5 cell blocks (Section 4.6.2). For an overview of data processing and analyses conducted herein we refer to Fig. 1.

## 4. Results and discussion

### 4.1. The relationship between built-up probability and built-up fraction

**Fig. 6.**
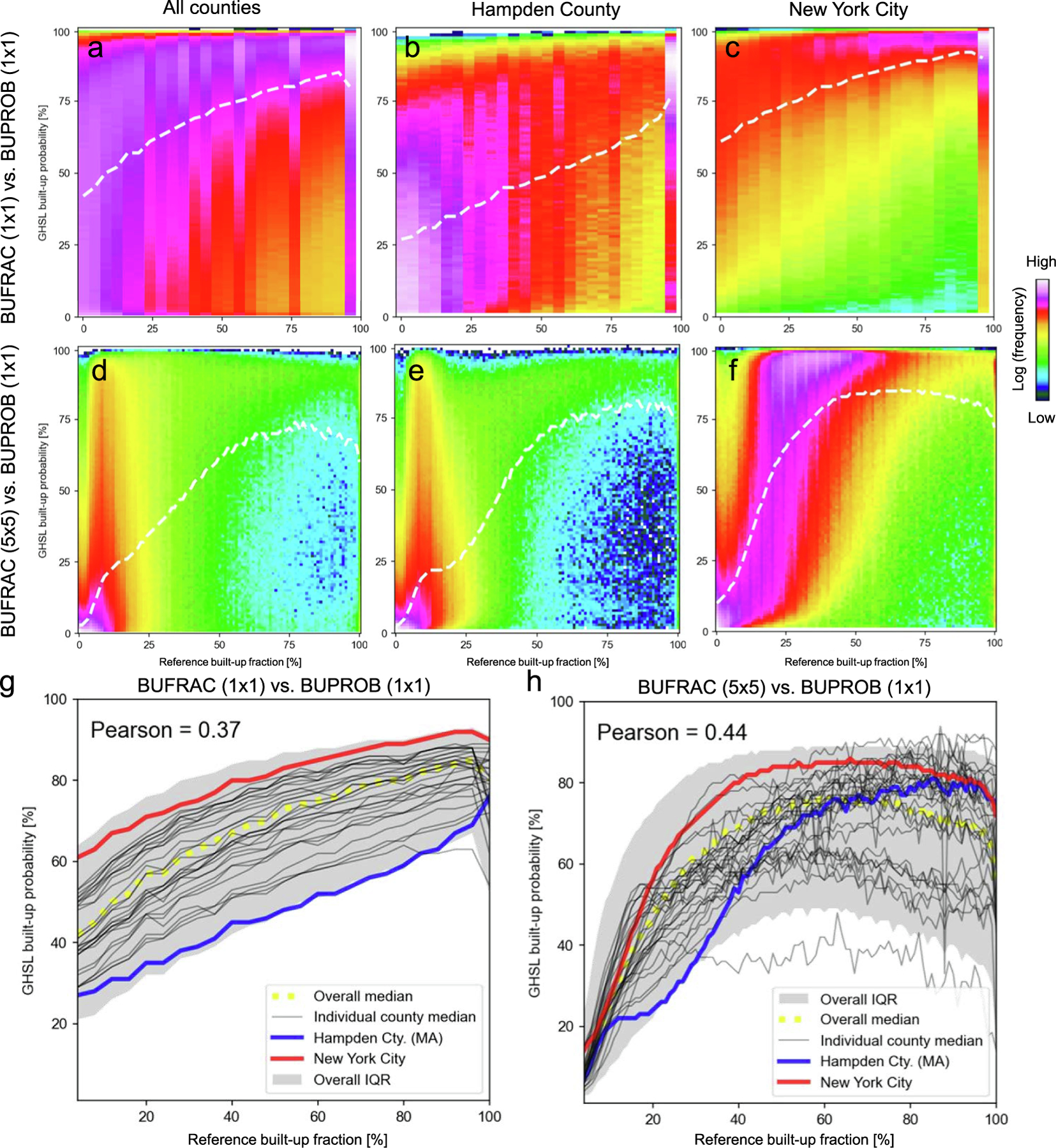
Assessing the relationship between built-up probability (BUPROB) reported in GHS-BUILT-S2 and built-up fraction (BUFRAC) derived from the reference data: Bivariate histogram of BUPROB and BUFRAC at the grid-cell level: (a) across all countries, (b) for Hampden County, (c) for New York City; bivariate histograms based on cell-level BUPROB and BUFRAC calculated in moving windows of 5 × 5 cells, to account for the spatial context used for CNN inference, for (d) all counties, (e) Hampden County, and (f) New York City; bottom row shows median trendlines of BUPROB in bins of BUFRAC for (g) 1 × 1-cell BUFRAC vs. 1 × 1-cell BUPROB, and (h) for 5 × 5-cell BUFRAC vs. 1 × 1-cell BUPROB.

**Fig. A1.**
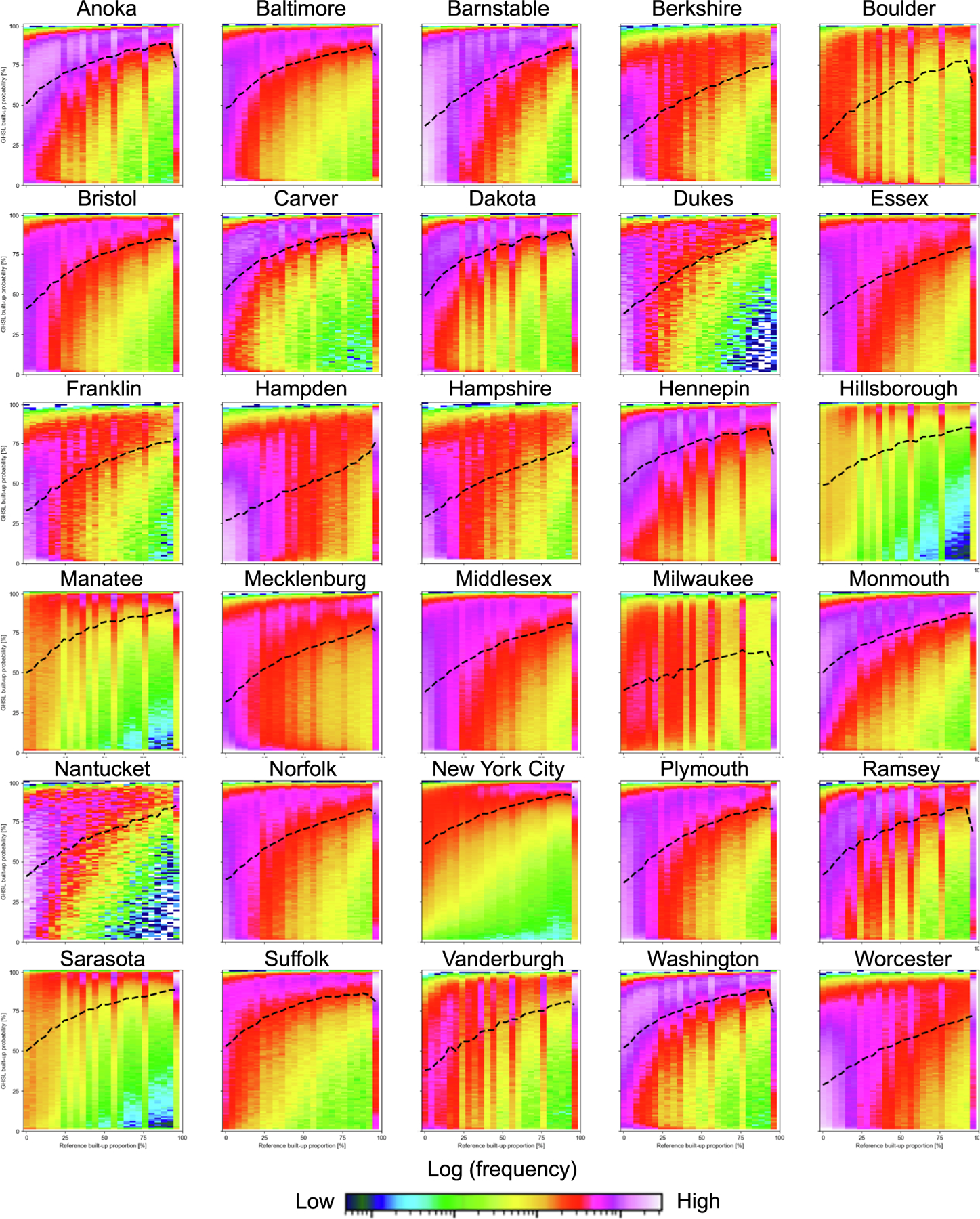
Bi-variate frequency distributions of built-up fraction (x-axis) and built-up probability (y-axis) for all 10 m grid cells within each of the 30 counties under study.

We binned the BUFRAC and BUPROB values for each county into bins of 4% and 1%, respectively, and visualized the bivariate histograms of the joint BUFRAC / BUPROB distribution. Moreover, we calculated the median BUPROB per BUFRAC bin. We observe generally a positive association between BUFRAC and BUPROB when using the data distribution over all counties (Fig. 6a), but we observe considerable variation in the bivariate histograms and median lines for individual counties (Fig. 6b,c, see Appendix Fig. A1 for all 30 counties under study). For example, we observe higher median BUPROB per BUPRAC bin in New York City (Fig. 6c) than in Hampden County (Fig. 6b). This effect can also be seen in the examples shown in Fig. 2, illustrating how large and densely built structures in New York City cause high levels of built-up probability, as opposed to smaller, less densely arranged buildings in Hampden County. This could also indicate that the classifier used to create the GHS-BUILT-S2 surface is more confident in detecting high-density structures as built-up, possibly also due to spillover effects caused by highly impervious surfaces in the vicinity of the buildings. Also, New York City may have been used as a training site for the GHS-BUILT-S2 classifier training phase, and the lower probabilities (which can be interpreted as lower levels of confidence) are the effect of weaker inference performance when generalizing on out-of-distribution data.

The county-level median BUPROB lines (Fig. 6g) indicate that even grid cells of very low BUFRAC have median BUPROB values of around 25% in Hampden County, and up to 60% in New York City. Thus, these two counties represent extreme cases among the counties under test. This is in line with the previous observations, and likely to be the result of spillover effects of impervious surface surrounding the buildings, as well as mixed-pixel effects in general, and the intensity of this effect is driven by the built-up density and the level of impervious surface. Another interesting observation is the drop of median BUPROB for grid cells of close to 100% built-up fraction in some counties. These are likely large buildings, possibly with a roof material that may occur more rarely in the training data, and this sparseness may cause lower levels of classification confidence.

Here, it is noteworthy that only grid cells have been taken into account where both BUFRAC and BUPROB are > 0, to avoid omission or commission errors to interfere in the analysis of the relationship between BUFRAC and BUPROB.

Despite the difference in the intercepts of the median BUPROB lines, the correlation coefficients between BUFRAC and BUPROB are relatively stable across counties, ranging from 0.25 to 0.4 for the majority of counties (see Appendix Table A1). The overall Pearson’s correlation coefficient between BUFRAC and BUPROB is 0.37, further confirming the generally observed (and expected) positive association of BUFRAC with BUPROB.

Can users infer the built-up fraction from the built-up probability at the cell-level? To address this, we used a curve fitting approach to estimate BUFRAC as a function of BUPROB. As can be seen in Fig. 6 a–c, a given BUFRAC value corresponds to a wide range of BUPROB values, and thus, this relationship is ambiguous. The patterns observed in Fig. 6 a–c suggest a non-linear relationship between BUFRAC and BUPROB, and thus, we fitted a range of polynomial functions of degree one to four using ordinary least squares. As suspected, the predictive power of BUPROB for BUFRAC is low, with R^2^ values rarely exceeding 0.2, and RMSE values of around 30% when estimating BUFRAC in % grid cell area (Appendix Table A2). These poor results indicate that built-up area fraction at 10 m resolution cannot be inferred reliably from the built-up probabilities provided by the classifier underlying the GHS-BUILT-S2.

**Fig. A2.**
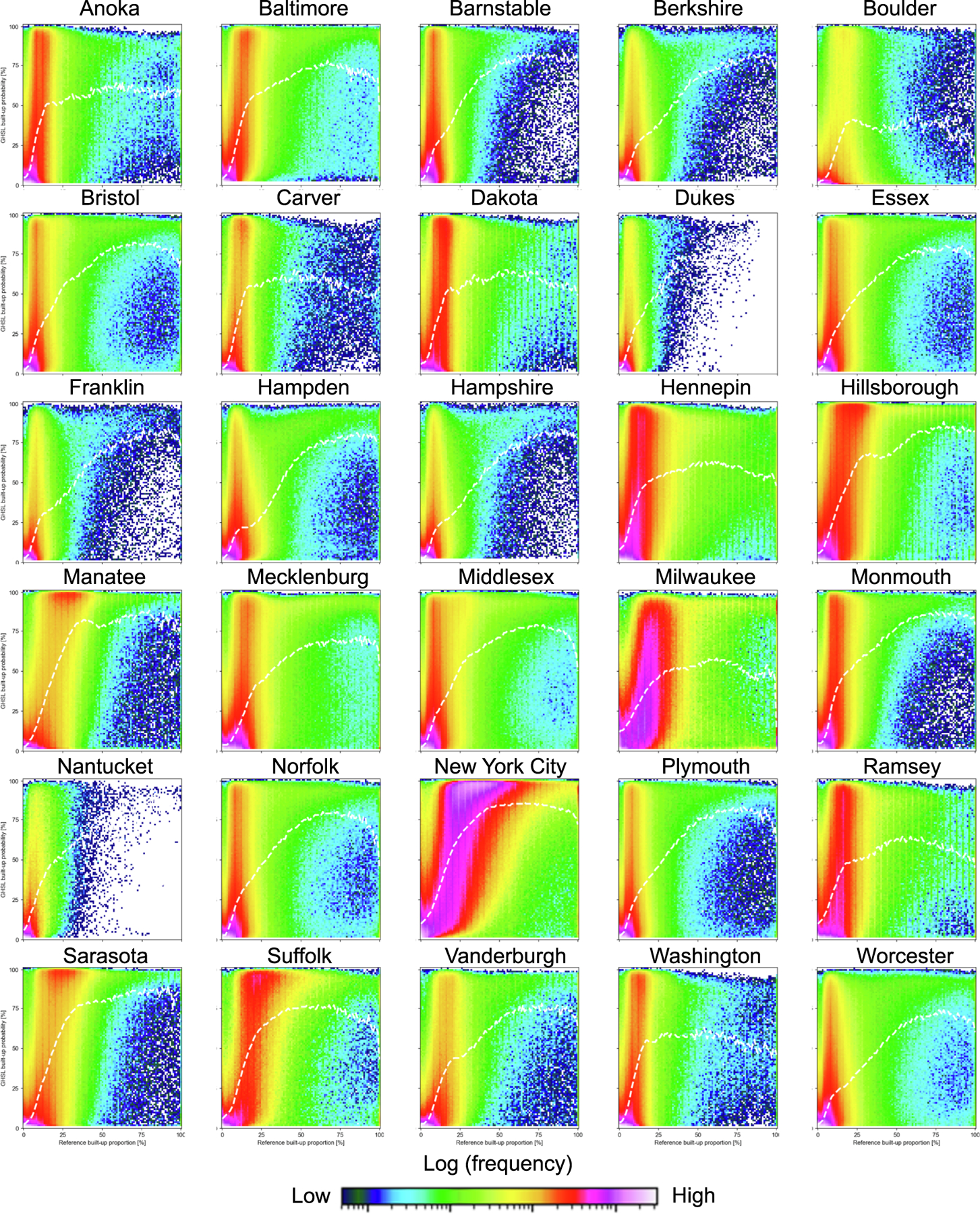
Bi-variate frequency distributions of built-up fraction (x-axis) and built-up probability (y-axis) for all 10 m grid cells within each of the 30 counties under study, using reference built-up fractions calculated within a moving 5×5-cell window, consistent with the image patches used as input for the GHS-S2Net classifier.

While these findings could be important for the interpretation of cell-level built-up probabilities and potential translation into built-up proportions, there is a slight scale mismatch between cell-level BUFRAC and BUPROB. According to Corbane et al. (2021) the CNN used to produce the GHS-BUILT-S2, (i.e., the GHS-S2Net; Corbane et al, 2021) uses image patches of 5 × 5 pixels of the underlying Sentinel-2 multispectral data. Thus, the CNN-based determination of the built-up probability at a given grid cell ***C*** is influenced by its spatial context, while the BUFRAC estimates in Fig. 6 a–c,g refer to the cell ***C*** only. Hence, to account for this mismatch, we calculated the BUFRAC within moving windows of 5 × 5 cells, to reduce this mismatch. The bivariate histograms of BUFRAC (5 × 5) and BUPROB (1 × 1) (Fig. 6,d–f) and the county-level median trendlines (Fig. 6h) show different patterns than their cell-by-cell level counterparts (Fig. 6a–c,g). Specifically, the previously observed intercepts are much smaller, indicating low levels of built-up probability for low levels of BUFRAC in the spatial window observed by the CNN. Moreover, BUPROB drops for samples of high BUFRAC in the 5 × 5-cell window, indicating lower levels of confidence of GHS-S2Net for large built-up structures extending across several grid cells. The high spread of BUPROB for grid cells of low BUFRAC (Fig. 6d,e) indicates lower levels of classification confidence for sparsely built-up regions. On average (Fig. 6h), highest built-up probabilities are obtained for 5 × 5-cell samples with a reference BUFRAC of around 60% to 70%, possibly indicating that the training data predominantly contained training samples with built-up fractions in that range. This additional assessment reveals interesting details regarding the way how the GHS-S2Net works: The wide range of BUPROB for similar, low values of BUFRAC across most counties (Fig. A2) indicates that features other than built-up fraction (or its manifestation in the Sentinel-2 multispectral data) are used as salient features within the GHS-S2Net for the determination of the probabilistic output.

### 4.2. Agreement between binary built-up / not built-up surfaces

**Fig. 7.**
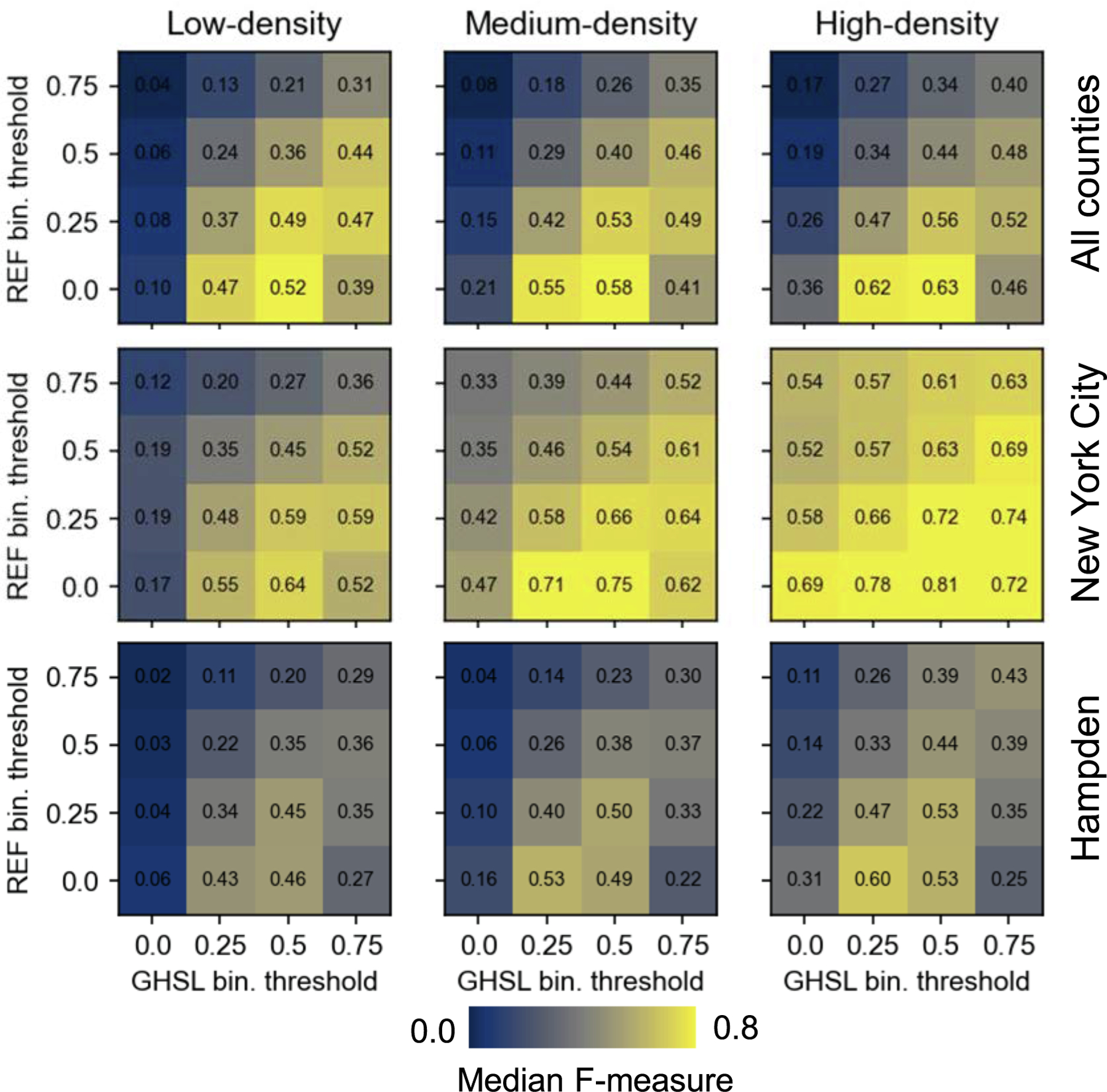
Binary agreement of boolean built-up / not built-up surfaces created from the GHS-BUILT-S1 and the reference data, by systematically applying different binarization thresholds to the continuous GHSL built-up probabilities and the built-up fractions derived from the reference data, shown across all counties under test, and for Hampden County (Massachusetts) and New York City.

When binarizing both, the continuous BUFRAC and BUPROB surfaces based on the cut-off values discussed in Section 3.2.2, we observe that the thematic agreement between these binarized surfaces is highest when using a threshold of > 50% for the built-up probabilities, and a threshold of > 0% for the reference built-up fraction surfaces. This observation is remarkably consistent across the three density-based strata (loosely related to rural, *peri*-urban, and urban areas), over all counties, for Hampden County and New York City (Fig. 7) and for most of the other counties under study (Table 1). In practice, this implies that by using a cutoff value of 50% applied to the GHS-BUILT-S2 surface, the resulting binary layer can be interpreted as a built-up area presence-absence surface, and this surface exhibits relatively high agreement with a binary surface that maps any grid cell containing a built-up area fraction > 0 as “built-up”. Specifically, the F-1 scores range from around 0.5 in the low-density strata to 0.65 or higher in the high-density strata. While these values were obtained at a native resolution of 10 m, the F-1 scores derived from 3 × 3 cell blocks (corresponding to 30 m × 30 m) increase in many counties to around 0.8 or higher, measured across all three strata (Table 1). These F-1 scores obtained at 3 × 3 cell blocks are comparable to results of accuracy assessments carried out in earlier work, using the Landsat-based GHS-BUILT-R2018A. However, these earlier experiments resulted in considerably lower F-1 scores across rural–urban gradients (cf. Leyk et al., 2018). This indicates that a remarkable jump in accuracy can be expected when using the GHS-BUILT-S2 as a basis for built-up area mapping, at least for the areas under study, likely for the US, and possibly elsewhere as well. Moreover, these findings are in line with accuracy estimates reported in Corbane et al. (2021). Here, it should be noted that while we found high levels of consistency of the agreement maximization threshold for our study sites in the U.S., these cut-off values are likely to be different in Europe or Asia, as indicated in Hafner et al. (2022) who identify an optimal threshold of around 0.5 for a study area in the U.S., and in Corbane et al. (2021), where a threshold of 0.2 (rural areas) and 0.5 (dense urban areas) is recommended to provide best results, globally, on average. Furthermore, the maximum agreement found for binarization thresholds of > 0% (BUFRAC) and > 50% (BUPROB) can be partially explained with the design of the training labels. According to the definition of “built-up area” in Corbane et al. (2021), any grid cell that overlaps with a roofed structure is considered “built-up”, corresponding to a threshold of > 0% built-up surface. Thus, this observation also underlines the good generalization capabilities of the relationship between training labels and Sentinel-2 data by the GHS-S2Net across different regions.

### 4.3. Spatially explicit accuracy assessment

**Fig. 8.**
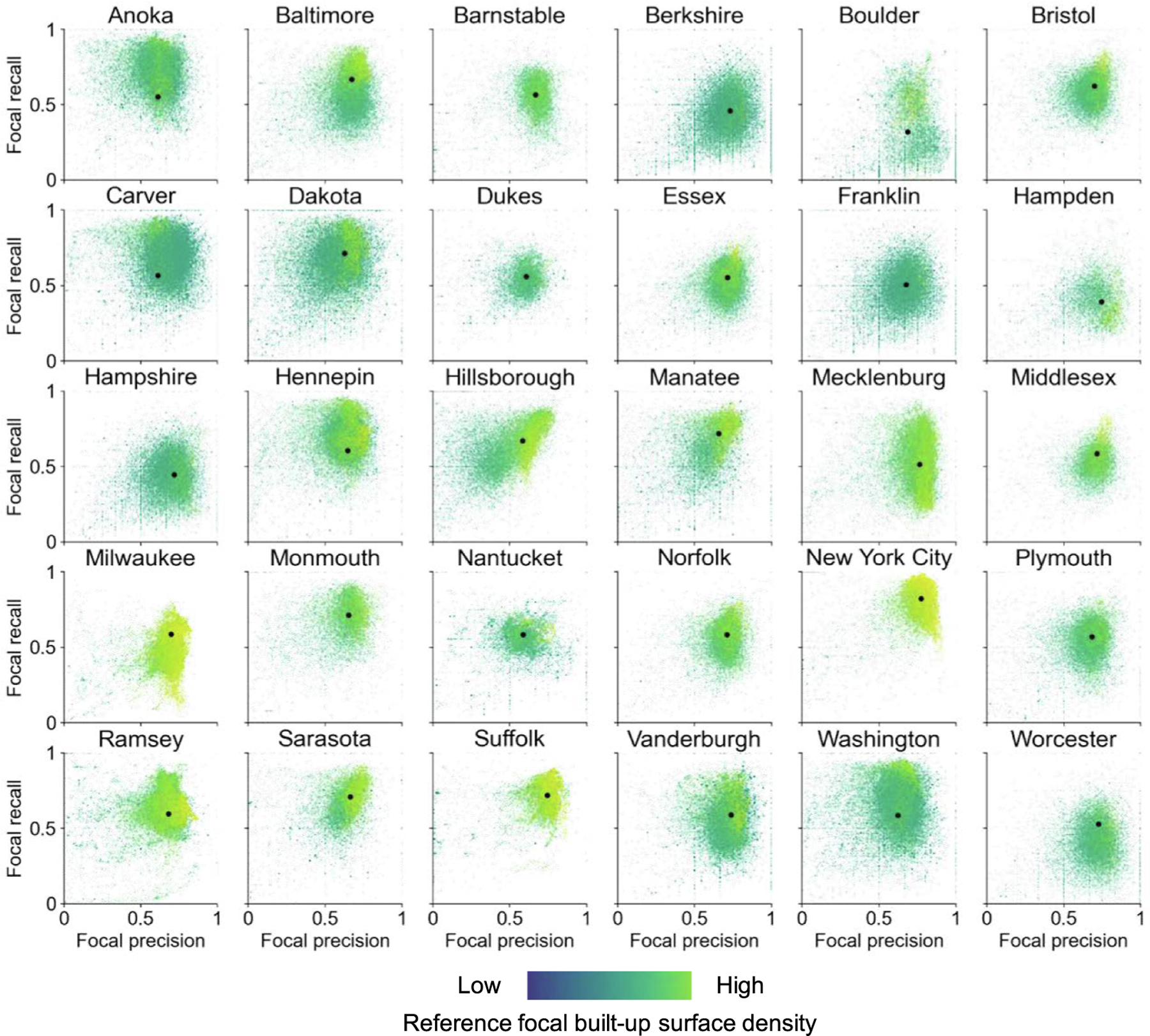
Focal precision-recall plots for all 30 counties, for a focal window size of 1 km. Coordinates of the black dots indicate precision and recall, respectively, calculated over the total county extents.

Based on the localized confusion matrices calculated in focal windows of 1 km × 1 km, we created focal precision-recall plots for each county (Fig. 8). The location, spread, and shape of the visualized point clouds provide rich information about the overall levels of commission and omission error, their variation within each county, and their interaction (Fig. 8). Thus, these plots represent a visual-analytical method to assess different aspects of map classification accuracy or agreement of binary surfaces within a given region, and to compare across regions. The additional color-coding of the data points indicating the reference built-up surface density within each focal window (scaled across all regions) enables the localization of specific commission-omission error combinations along the rural–urban continuum.

For example, New York City and Suffolk County (i.e., the city of Boston, Massachusetts) are among the counties of very high density, exhibiting high levels of completeness (i.e., recall) and correctness (i.e., precision) across their spatial extents. Surprisingly, Milwaukee County (i.e., the city of Milwaukee, Wisconsin) has similar levels of built-up surface density, but shows lower levels of precision, and even more pronounced, of recall. Mecklenburg County (i.e., the city of Charlotte, North Carolina) shows high levels of precision, but also higher levels of omission errors. Other point cloud shapes such as for Hillsborough or Sarasota County, and to a lesser degree, for Manatee County (all located in the Greater Tampa region, Florida), we see a more linear relationship of precision and recall, indicating that in places where the binarized GHS-BUILT-S2 surface is more correct, it is also more complete. This could also indicate that data from Florida was used for training the classifier underlying GHS-BUILT-S2, or the detection of settlements in that region is more straightforward than in others, possibly due to vegetation and other factors.

These plots also reveal other interesting cases, such as Boulder County (Colorado), which seems to be divided into a high precision and high recall region (likely the city of Boulder itself), and places of high precision but low recall (possibly the scattered, rural settlement in the Mountains and the Plains). Counties with data points spread towards the left part of the graphs (i.e., low levels of precision) could be affected by recent growth in built-up area (during 2015 to 2018) that is not contained in the MTBF-33 reference data (dated to 2015 or earlier in some counties), but correctly measured in the GHS-BUILT-S2 (reflecting the state of built-up areas in 2018). Thus, these locations could contain higher levels of false positives, induced by missingness in the reference data. Finally, the black dots illustrating the overall precision-recall pairs calculated across all grid cells per county once more illustrate the need for spatially explicit accuracy assessments, as overly generalized accuracy metrics often fail to take into account the spatial non-stationarity of the uncertainty in geospatial data (e.g., Leyk and Zimmermann, 2004, Strahler et al., 2006, , Foody, 2007, Wickham et al., 2018).

**Fig. 9.**
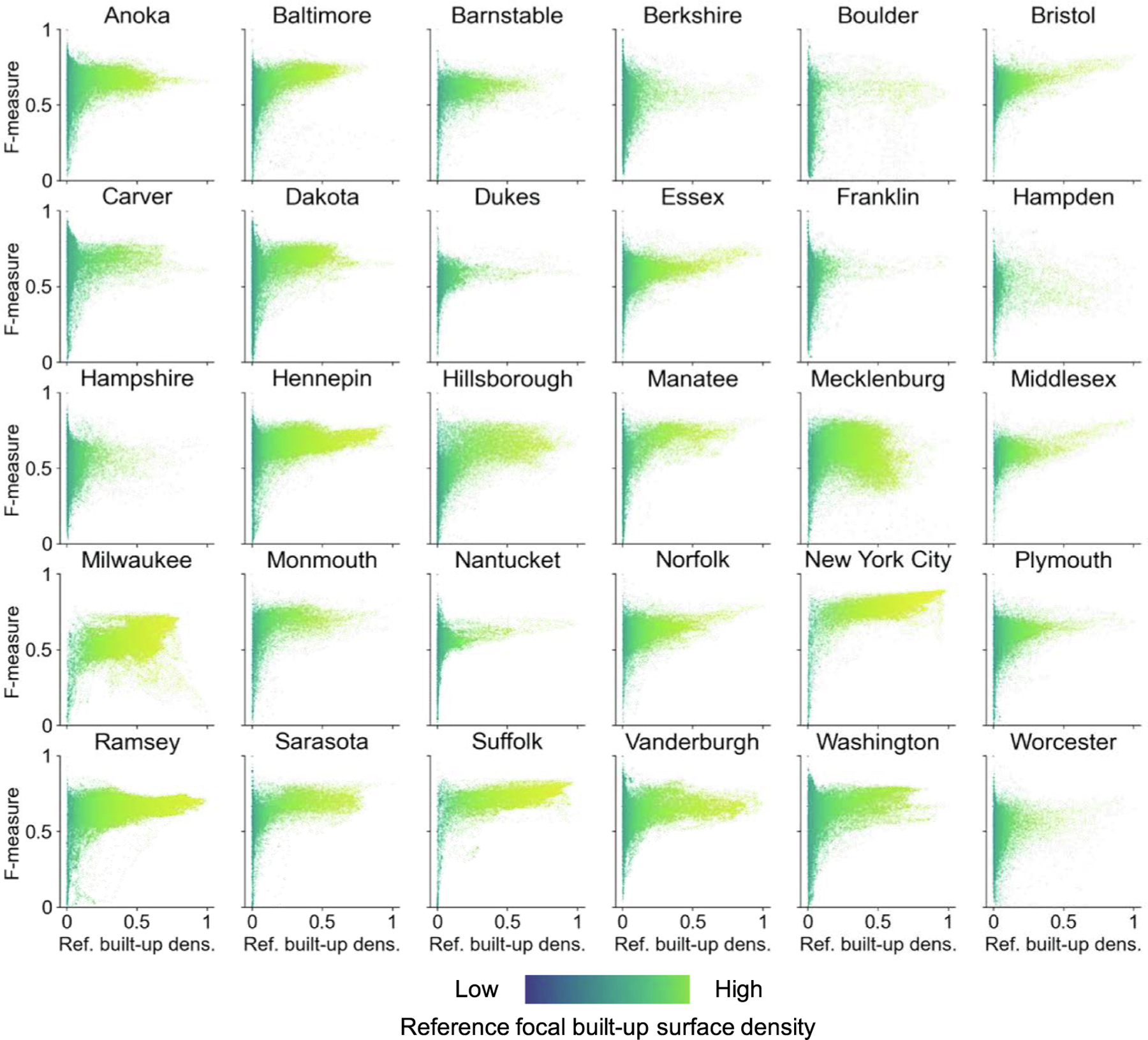
Focal accuracy estimates along the rural–urban continuum: Relationship between F-measure and reference built-up density per county, for a focal support of 1 km.

We also used our focal accuracy estimates to assess the accuracy variations along the rural–urban continuum. While there are multiple ways to model the gradient from rural to urban areas (e.g., Waldorf & Kim, 2015), we used the built-up surface density derived from the reference data, as it is enumerated consistently to our focal accuracy estimates (see Section 3.2.3). We did this visually-analytically by transforming the reference built-up densities into the range [0,1] and plotting them against the F-1 score computed for each focal window (Fig. 9). These plots indicate that low accuracy (as measured by the F-1 score) almost exclusively occur in low-density regions. F-1 levels then increase steadily at low slope towards high-density regions. In some cases, F-1 scores slightly drop towards the high-density regions, likely due to mixed-pixel effects and spillover effects of impervious surfaces, in highly built-up areas, resulting in higher levels of commission errors. Generally, these trends are very encouraging, as compared to similar assessments of earlier versions of the GHS-BUILT, where the trends across the rural–urban continuum were much steeper, indicating an improved mapping of rural and *peri*-urban settlements in the GHS-BUILT-S2 dataset and less of a difference in data quality between rural and urban settings. We summarize these accuracy trends across different GHSL data versions in Table 2.

### 4.4. GHSL built-up area accuracy trends across data versions

Putting the overall results reported herein in the context of previous evaluation studies consistently based on the MTBF-33 dataset, we observe two interesting trends: 1) mapping accuracy (as measured by the F-1 score) increases in rural areas from 0.11 (GHS_LDSMT_2015) to 0.52 (GHS-BUILT-S2), whereas it decreases in urban areas from 0.85 (GHS_LDSMT_2015) to 0.63 in GHS-BUILT-S2 (Table 2). While it is very promising to observe that the detection of rural settlements has increased considerably across versions, as a result of higher data resolution and improved information extraction strategies, the decrease in the F-1 score in urban settings may be due to the finer resolution of the data: At lower spatial resolutions, F-1 is higher due to an aggregation effect (i.e., built-up areas may also encompass roads between buildings), whereas at finer resolution (10 m, 20 m), individual buildings are mapped, and the spaces between buildings are labelled as not built-up. This difference in granularity may cause higher levels of both omission and commission errors, particularly in semi-dense urban settings characterized by small buildings and thus, result in lower F-1 scores in urban settings overall. In the latter case, the effects of positional uncertainty on thematic accuracy may also have a larger effect. This becomes evident when looking at Table 1, where the F-1 scores calculated in 3×3-cell windows often exceeds 0.8, and yielding an average F-1 score of 0.75. In summary, we can say that across data versions, accuracy in rural areas has increased, and the accuracy gradient between rural and urban areas has become less steep.

### 4.5. Regression analysis

While the assessment presented in Section 4.3 focused on thematic agreement of built-up vs. not built-up grid cells, we also assessed the quantity agreement of built-up surface within our focal windows, by means of regression analyses, relaxing the constraint of spatial coincidence of built-up grid cells. Using a linear regression model to estimate reference built-up quantity based on the GHS-derived built-up quantity (measured within the 1 km × 1 km focal windows, ranging from 0 to 1), we find a highly linear relationship with an R^2^ of 0.93 across all counties, a slope of 0.87 and an intercept of 0.20 (Table 3). These regression models perform similarly at the county level, with R^2^ values of > 0.9 in most counties. The slope values, however, exhibit some variations across counties, with a minimum value of 0.44 in Hampden County. As we have seen in Fig. 6, Hampden County is also the county with the lowest built-up probability, on average.

**Fig. 10.**
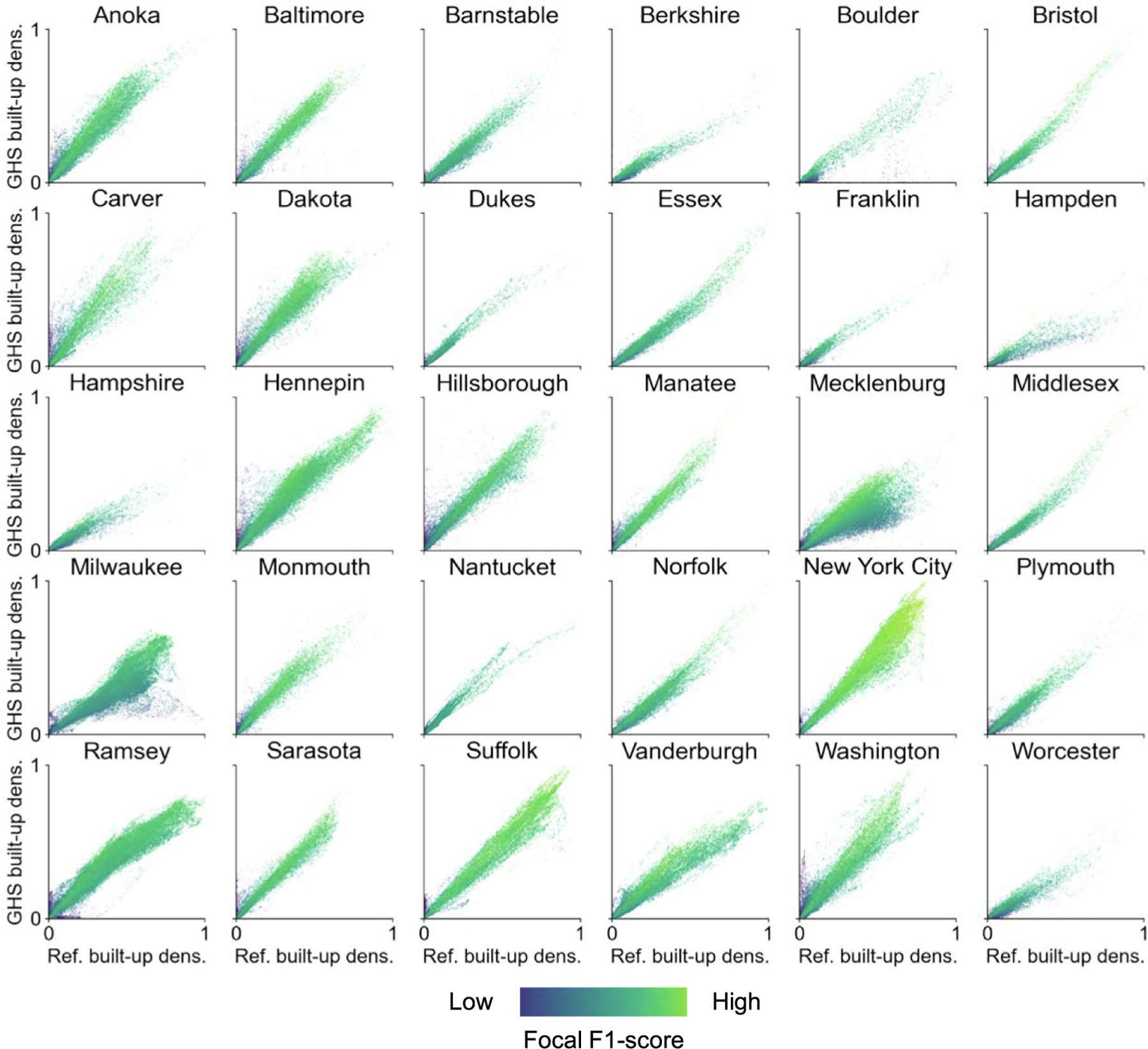
Scatterplots of reference and GHSL-based built-up quantity per county for a focal support level of 1 km.

Thus, the observed differences in the relationship between built-up quantities are likely an effect of lower levels of classification confidence in the GHS-BUILT-S2 in some regions. The scatterplots of focal built-up quantity from the GHSL and the reference data confirm these observations (Fig. 10). In some counties we observe superposed effects of a linear relationship for low and medium density areas, and a superlinear relationship towards high-density areas, where the built-up quantity in GHS-BUILT-S2 exceeds the reference built-up quantity (e.g., Bristol, Essex, Middlesex counties). The opposite trend can be observed for Suffolk County (i.e., the city of Boston), where in high-density areas the relationship becomes sublinear.

### 4.6. Sensitivity analysis

As noted earlier, the outcomes of thematic accuracy assessments based on gridded data may be biased by positional uncertainty in the underlying spatial data, that can result in random or even systematic misalignment of grid cells. This issue is addressed in Section 4.6.1. Moreover, the analytical unit at which the assessment is conducted, may also affect the results, due to the same misalignment but also general aggregation issues, subject to the Modifiable Areal Unit Problem (MAUP; Openshaw and Taylor, 1979, Flowerdew et al., 2001, Goodchild, 2022). Finally, the level of spatial support (i.e., the focal window size used to calculate localized accuracy estimates) may affect the results, which is another manifestation of the MAUP. Hence, we systematically varied both the analytical unit and the spatial support to assess the effects on our focal precision and recall estimates (Section 4.6.2).

#### 4.6.1. Sensitivity to spatial offsets

**Fig. 11.**
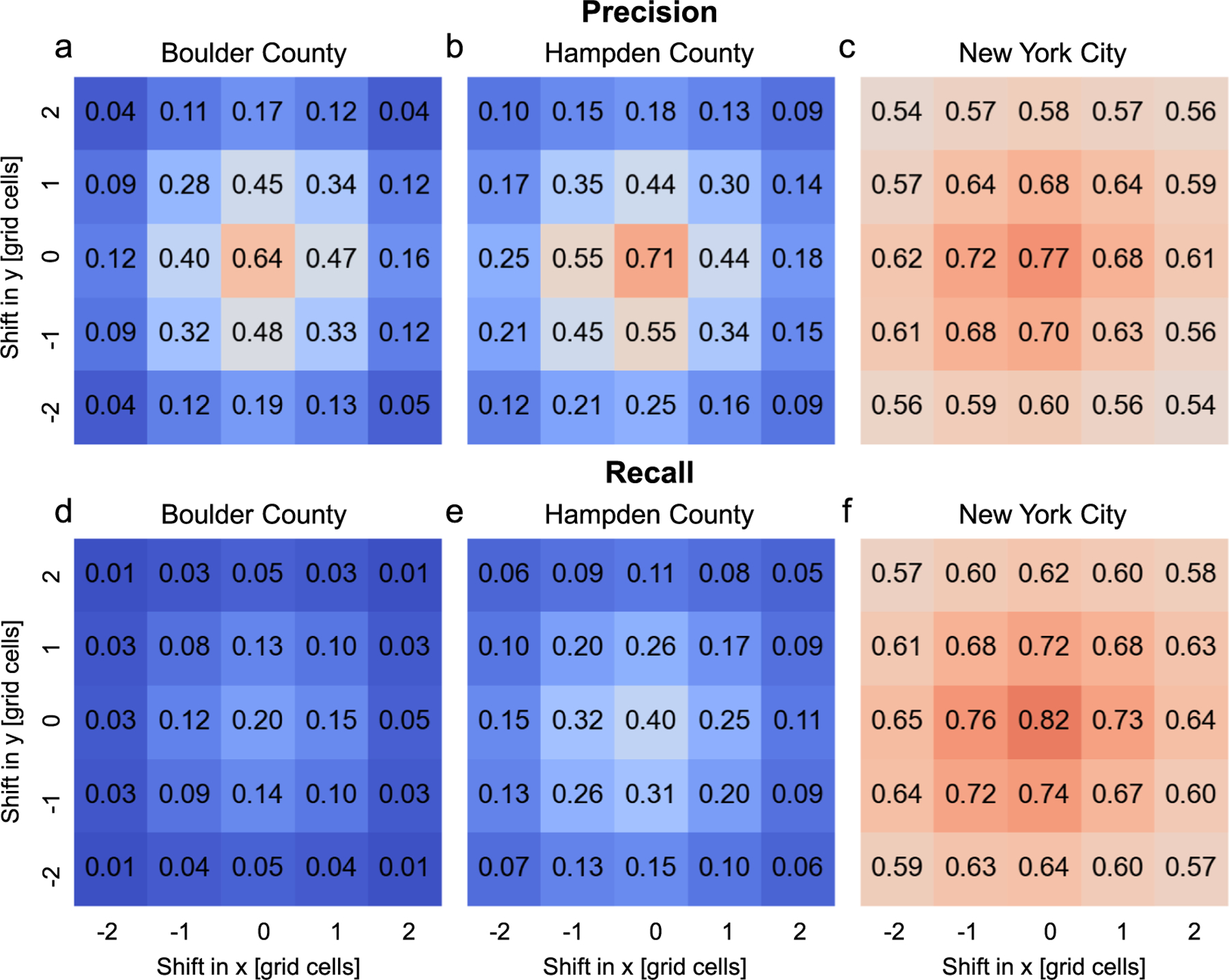
Sensitivity of focal (thematic) accuracy estimates to spatial offsets, modelled by systematically shifting the reference and test grids. Average focal precision in (a) Boulder County, (b) Hampden County, and (c) New York City, and (d-f) corresponding plots for average focal recall, based on 24 combinations of shifts in x and y direction, applied to the reference data.

We systematically shifted the reference grid by 1 and 2 grid cells in each direction, and recomputed the focal precision and recall values for each combination of grid shifts in x-and y-direction. To keep the computational effort to a feasible level, we only conducted this analysis for three counties (i.e., Boulder County, Hampden County, and New York City). Fig. 11 reports the average focal precision and recall for each shift combination. We observe a sharp drop of average precision and recall, even if grids are shifted by 10 m only. This effect is most nuanced in Boulder County, where this drop from 0.64 to around 0.45 for precision (Fig. 11a), and from 0.2 to 0.13 for recall (Fig. 11d), corresponds to relative decreases in average agreement (i.e., approximately 27% for precision, and approximately 45% for recall, respectively). This effect is least dominant in the highly urban study area of New York City (Fig. 11 c,f; around 10% relative drop when shifting grids by 10 m), which can be explained by the spillover and mixed-pixel effects occurring in highly impervious areas. This stark contrast between high-density settings and rural counties (i.e., Boulder County is largely characterized by scattered, rural settlements in the Mountains and the Plains) emphasizes the importance of the positional accuracy of spatial data as a prerequisite for unbiased thematic accuracy assessments.

**Fig. 12.**
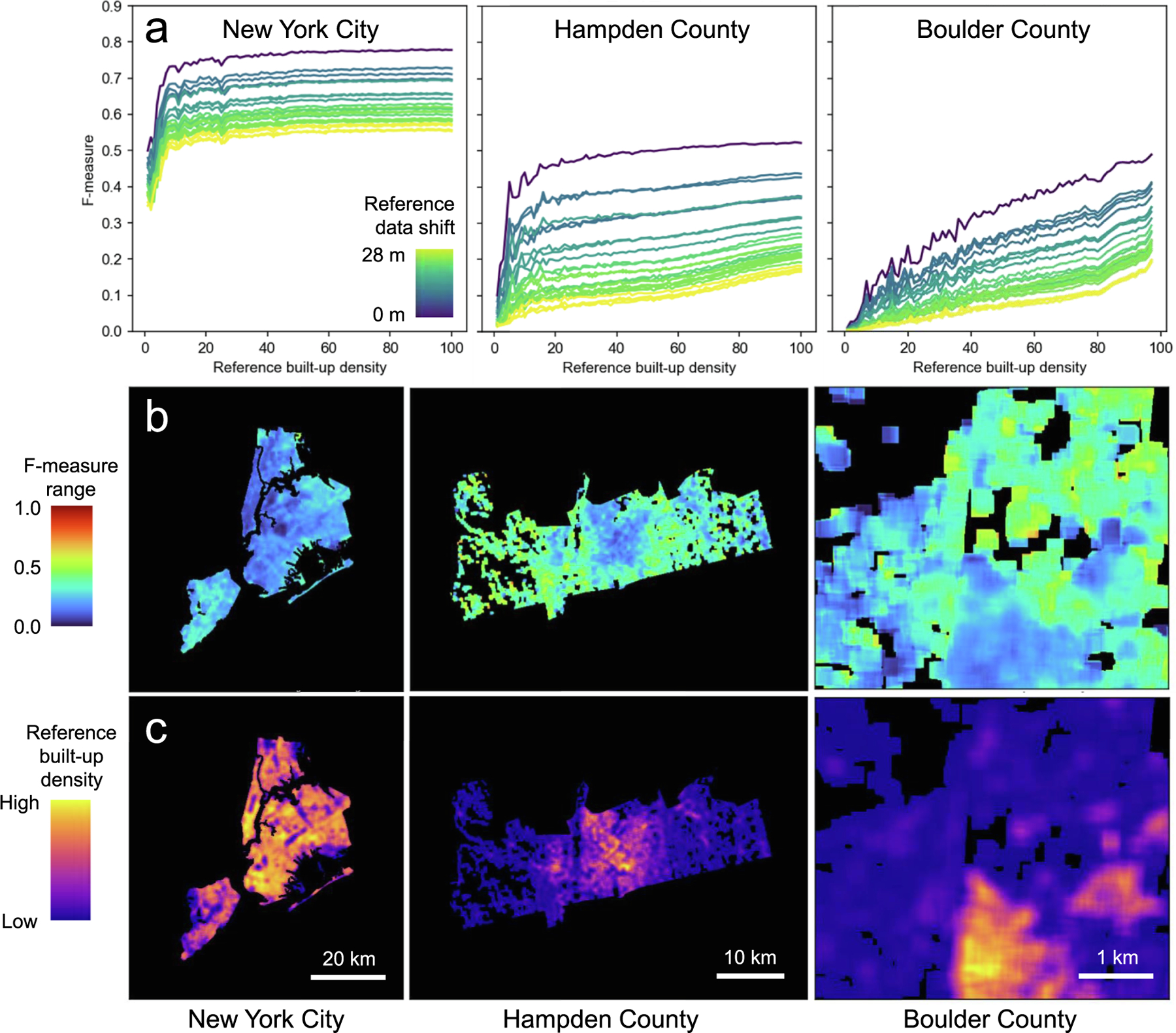
Sensitivity analysis of the focal accuracy measures to systematic offsets between GHSL and reference data: (a) median trendlines of F-1 score per reference built-up area density stratum (using 100 equal-width bins), (b) maps of the range of focal F-1 scores per grid cell, based on the 25 grid shift combinations, and (c) the reference built-up surface density for comparison, illustrating that F-1 score ranges are narrower in high-density regions. All data shown for New York City, Hampden County (Massachusetts), and for a subset of Boulder County (Colorado).

While these shifts appear to affect the overall level of accuracy estimates as a function of the settlement density in a given region, their effect on the accuracy trajectories is less nuanced (Fig. 12a): While the trend lines are shifted along the y-axis (as a result of lower agreement), their slopes along the rural–urban continuum remain largely unaffected, except for areas of extremely low settlement density in Hampden and Boulder counties. Finally, we visualized the range of F-1 scores at the grid cell level, for each of the 25 grid shift scenarios (Fig. 12b). These maps reveal further detail about our previously found relationship between settlement density and accuracy sensitivity: Even within our three test counties, the sensitivity of accuracy estimates to positional offsets varies, and these variations exhibit an inverse trend to settlement density (Fig. 12c), with lowest F-1 score dispersion levels found in high-density areas within a county.

These results illustrate that accuracy estimates are differently affected by positional uncertainty in the underlying spatial data. As we can generally assume that geospatial data quality is higher in high-density urban areas than in rural regions, which may also affect our reference data (e.g., terrain variations, occlusions from vegetation, less frequent data update cycles in rural areas), it is reasonable to assume that our accuracy estimates in rural areas may be negatively biased by such positional inaccuracies, and that the “true” accuracy of the GHS-BUILT-S2 data in rural areas is even higher than reported herein.

#### 4.6.2. Sensitivity to the spatial support and to the analytical unit

**Fig. 13.**
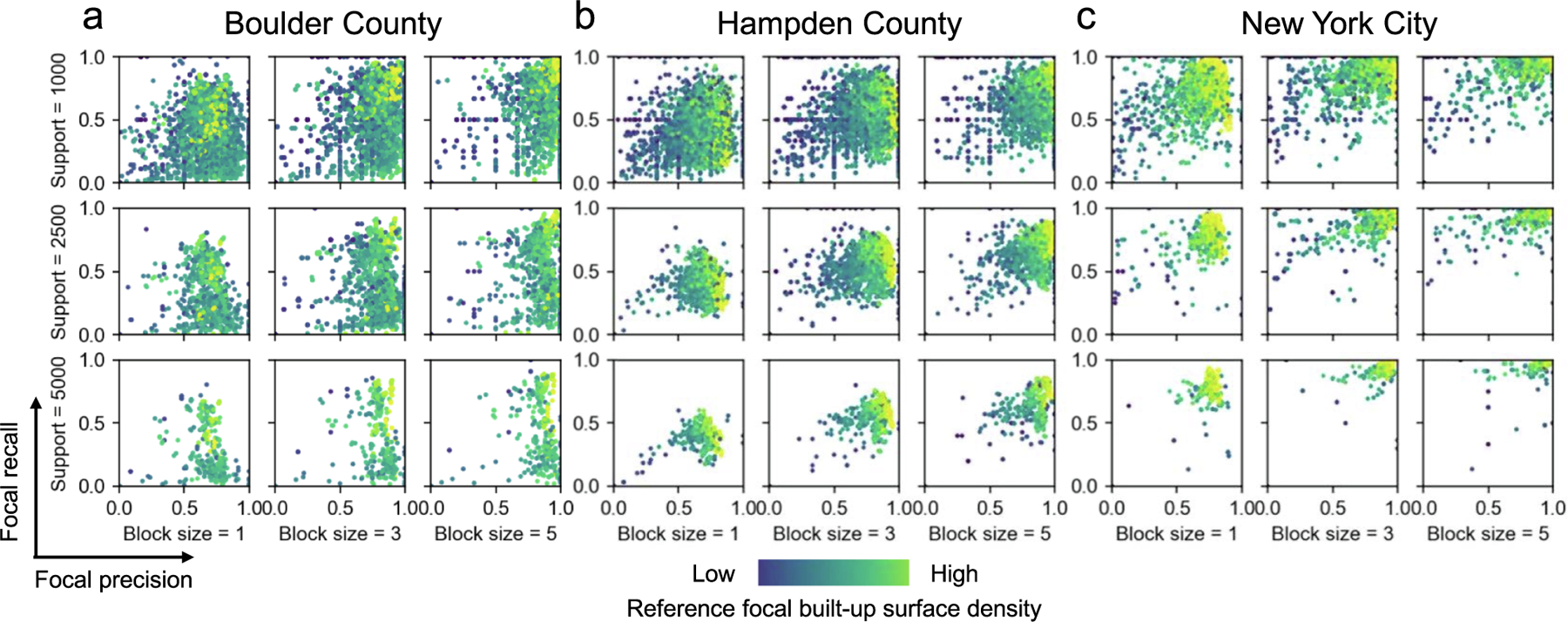
Sensitivity of focal precision-recall plots to analytical scale and spatial support. Shown are focal precision-recall signature plots with varying analytical unit (i.e., block size from 1 × 1 grid cells, 3 × 3 grid cells, and 5 × 5 grid cells) in x-direction, and varying spatial support (i.e., focal window size, ranging from 1 km (top row) to 5 km (bottom row).

**Fig. A3.**
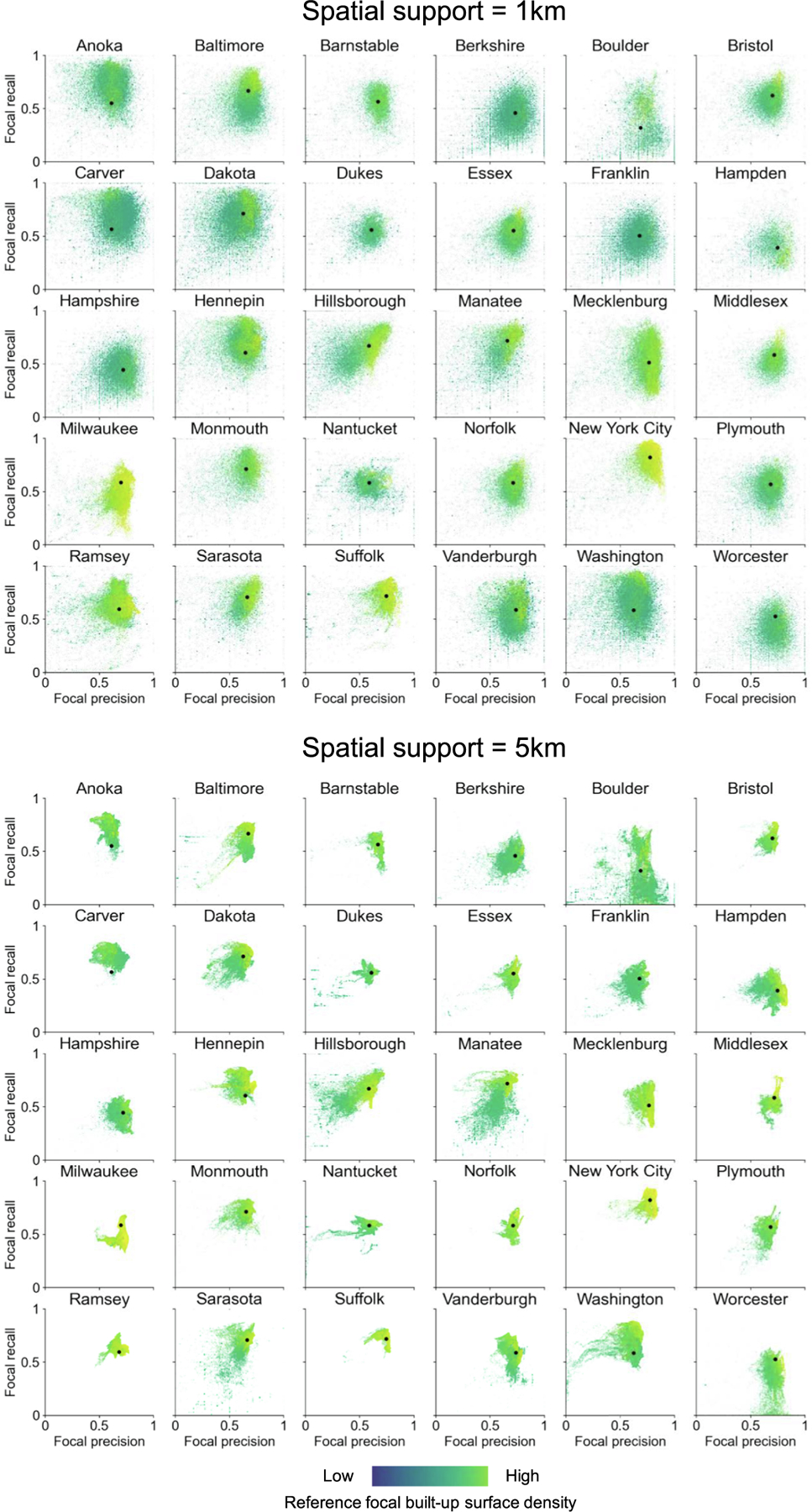
Focal precision-recall signature plots for a spatial support of 1 km and 5 km.

**Fig. A4.**
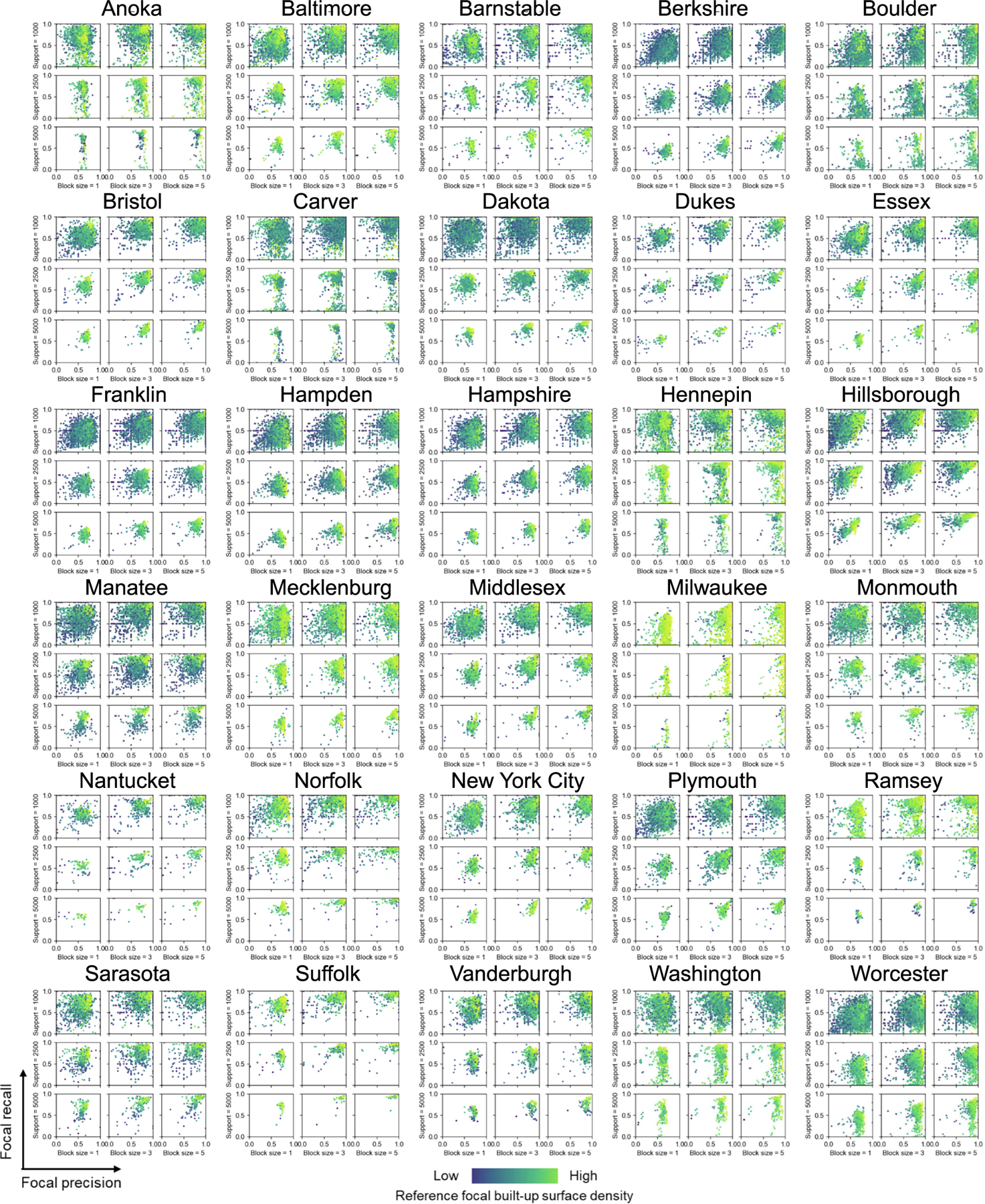
Scale sensitivity analyses per county. Shown are focal precision-recall signature plots, with varying analytical unit (i.e., block size from 1 × 1 grid cells, 3 × 3 grid cells, and 5 × 5 grid cells) in x-direction, and varying spatial support (i.e., focal window size, ranging from 1 km (top row) to 5 km (bottom row).

**Fig. A5.**
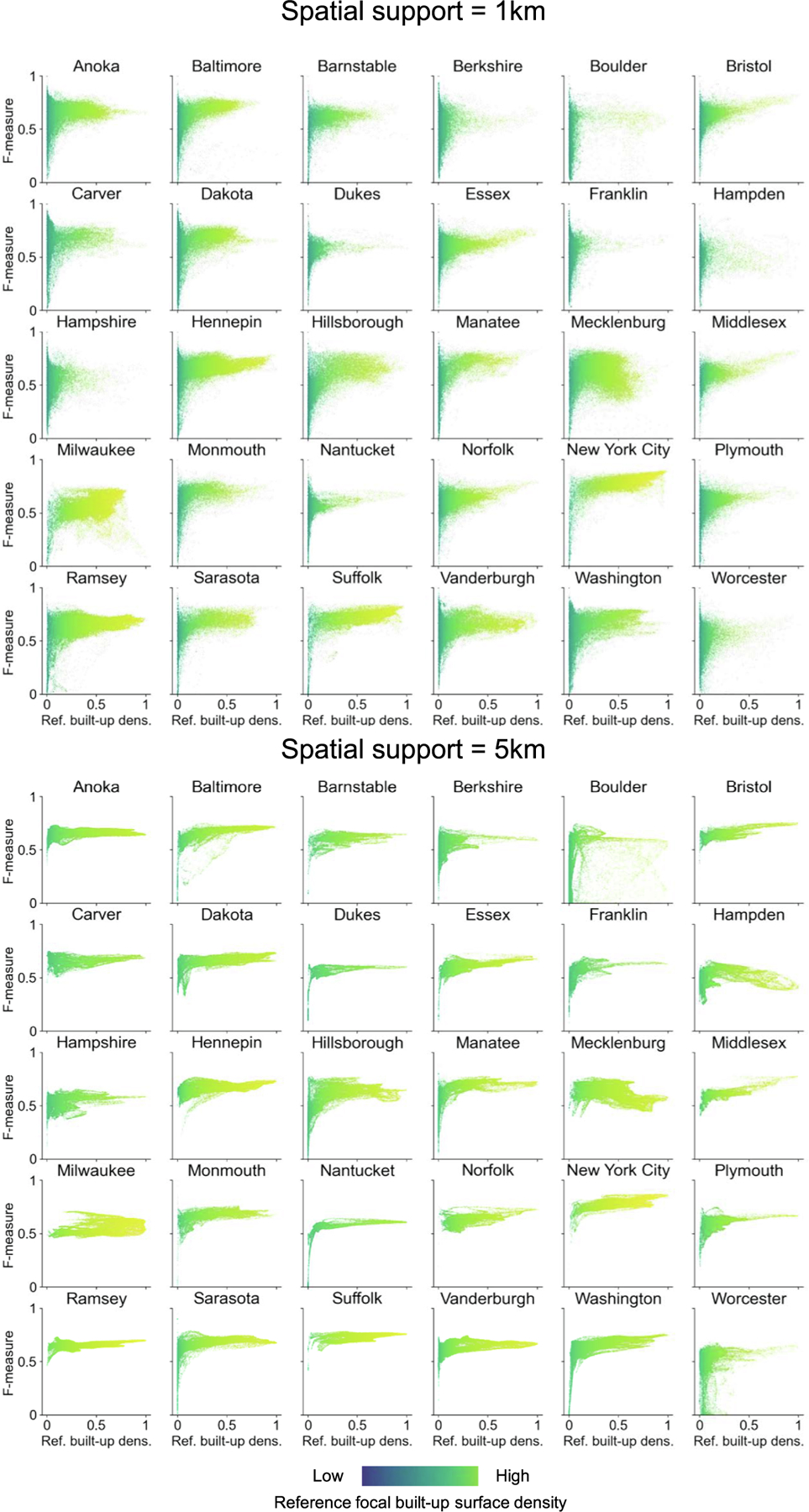
Focal built-up density vs. F-measure plots for a spatial support of 1 km and 5 km.

Lastly, we tested the effect of increasing block size (i.e., analytical unit) and increasing spatial support (i.e., focal window size used as analysis extent) on our focal precision-recall signature plots (cf. Fig. 8). When increasing the block size, we observe increasing accuracy, in both precision and recall (Fig. 13). This increase in the analytical unit is a mechanism to account for slight offsets in the gridded data, which would cause disagreement when calculated at the native resolution. We observe indeed increasing levels of both, precision and recall, in low-density but also high-density regions (Fig. 13). Interestingly, increasing spatial support leaves overall precision and recall levels largely unaffected, but the dispersion of the focal accuracy metrics is reduced as we increase the spatial support, which is a general data aggregation effect. The same can be observed for the focal precision-recall signature plots calculated for a support of 5 km, for all 30 counties (Fig. A3), and jointly for increasing support and block size (Fig. A4). A similar effect is also notable when visualizing F-1 scores trajectories across the rural–urban continuum (Fig. A5): Extreme values disappear due to the increased aggregation of the focal accuracy estimates. Hence, it is recommended to keep the focal window size small – large enough to ensure a robust sample size, and small enough to capture the spatial details in accuracy variation, as a large focal window size occludes fine spatial details in accuracy variation, and also increases the computational effort (i.e., more grid cells to take into account for each accuracy metric computation).

The increase of the analytical unit not only affects localized (focal) accuracy estimates, but also global (i.e., county-level accuracy estimates), exhibiting a similar trend, and potentially representing more realistic accuracy estimates (Table A3).

**Fig. A6.**
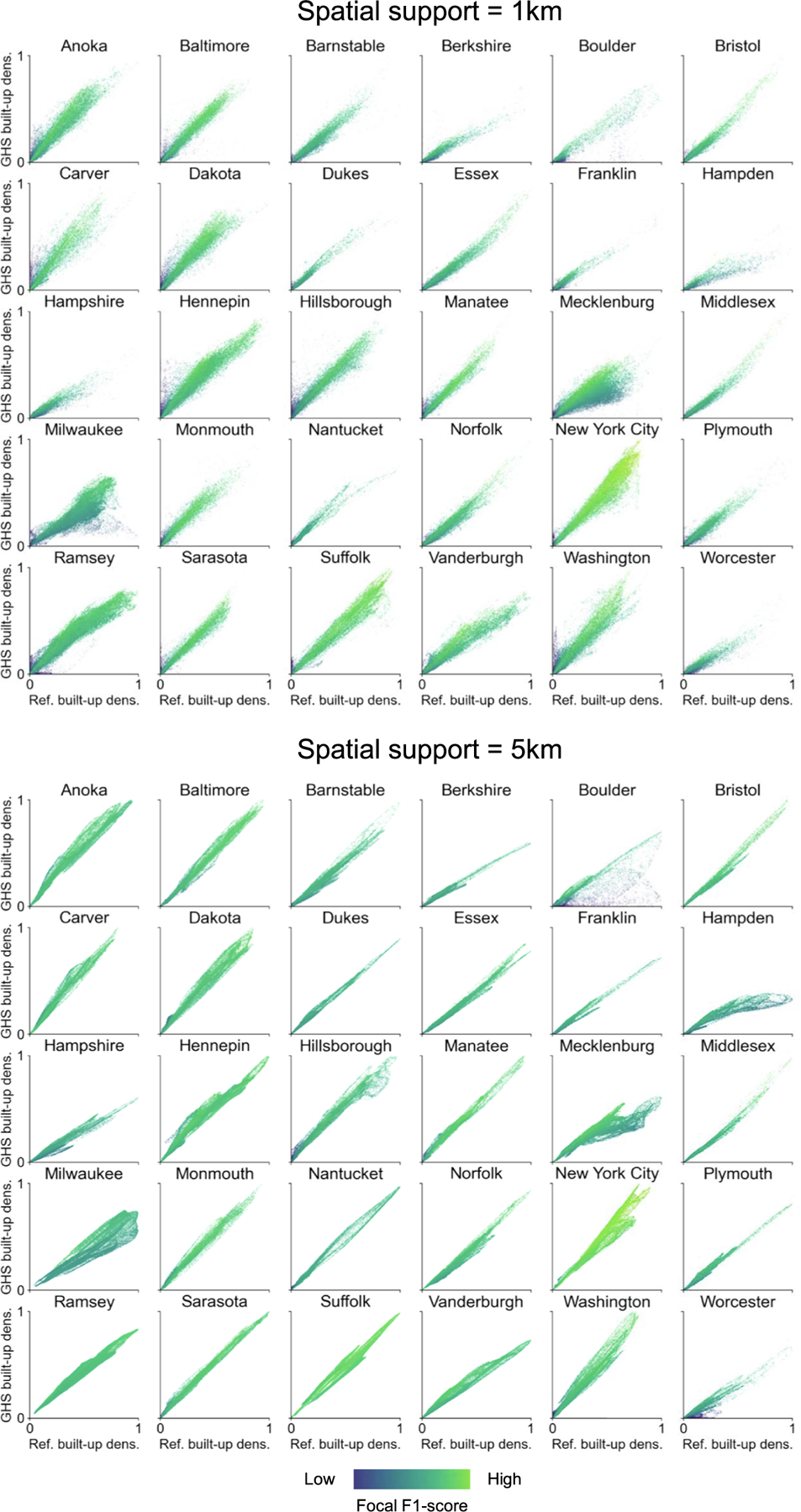
Focal reference built-up quantity vs. GHS built-up quantity per county, for a spatial support of 1 km and 5 km.

Finally, we also tested the effect of spatial support on the quantity agreement analysis (cf. Section 4.4). When comparing the regression analysis results of focal built-up density across different levels of spatial support, we observe increasing R^2^ values as we increase the spatial support (an effect of extreme values being aggregated, see Fig. A6, and thus, reducing the impact of their residuals on the R^2^), but we also observe fairly stable regression coefficients across different support levels (Table A4), indicating that the relationship between the reference and test built-up density is largely unaffected by the choice of the spatial support.

## 5. Conclusions

Herein, we presented a framework for the accuracy assessment of high resolution (i.e., 10 m) probabilistic built-up surface indicators. Specifically, we used machine learning-based probabilities of built-up area presence, as reported in the GHS-BUILT-S2 dataset and developed a multi-stage strategy for their evaluation against highly reliable building footprint data. The first stage is the analysis of the relationship between built-up probability and reference built-up fraction. While we found positive associations between these two variables, we also found that built-up probability cannot be directly translated into built-up area fractions, as indicated by the poor regression model performance (Section 4.1). In the second stage, we binarized both BUPROB and BUFRAC surfaces, and assessed for which binarization threshold the agreement between these binarized surfaces maximized. We found that, for a threshold of 50%, applied to the GHS-BUILT-S2 data, the resulting binary built-up presence-absence layer shows the highest agreement with the reference dataset, which labeled any grid cell as built-up if it contains at least one building (or a part of it). This thresholding approach appeared to be an effective way to interpret and translate machine learning-based probabilities into a meaningful physical measure. In the third stage, we assessed the thematic and quantity agreement of the resulting built-up area layers and observed increasing accuracy from rural to urban settings, but we also observed much higher levels of accuracy (both precision and recall) in rural areas, when compared to earlier versions of the GHS-BUILT data.

While this multi-stage framework can be applied to similar datasets, we also proposed a novel visual-analytical tool for spatially explicit accuracy assessments of binary gridded data, which are focal precision-recall signature plots (cf. Section 4.3). We also highlighted multiple methods for analyzing the sensitivity of accuracy estimates. Knowledge of the accuracy of the GHS-BUILT-S2 provides important guidance for an unbiased and informed interpretation of the dataset itself, and for follow-up data products such as the GHS-BUILT R2022A that are partially based on the GHS-BUILT-S2 (Schiavina et al., 2022).

A minor shortcoming of our analysis is the temporal gap between the MTBF-33 reference dataset (referred roughly to 2015) and the GHS-BUILT-S2 from 2018. The urban growth from 2015 to 2018, not measured by MTBF-33 would result in lower precision values. However, as we observe high precision levels throughout our analysis, we believe that this temporal discrepancy has only a minor effect on our findings. In future work, we will test different methods for the agreement maximization stage (e.g., ROC analysis; Green & Swets 1966), and apply this framework to larger spatial extents, within and outside of the United States.

## Figures and Tables

**Table 1:** Agreement-maximization threshold for Reference data and GHSL, across different strata of urbanness, and corresponding F-1 scores per stratum, global (i.e., per county) at full resolution, and global within 3×3 cell blocks.

	Agreement maximization threshold						F-1 score				
Stratum	Low-dens.		Medium-dens.		High-dens.		Low-dens.	Medium-dens.	High-dens.	overall 1×1	overall 3×3
County	Ref.	GHSL	Ref.	GHSL	Ref.	GHSL					
Anoka County	0%	50%	0%	50%	0%	50%	0.612	0.659	0.667	0.581	0.715
Baltimore County	0%	50%	0%	50%	0%	50%	0.507	0.575	0.669	0.671	0.804
Barnstable County	25%	50%	0%	50%	0%	50%	0.473	0.587	0.617	0.613	0.803
Berkshire County	0%	50%	0%	25%	0%	25%	0.533	0.536	0.590	0.565	0.706
Boulder County	0%	25%	0%	25%	0%	25%	0.462	0.455	0.554	0.436	0.571
Bristol County	0%	50%	0%	50%	0%	50%	0.538	0.600	0.640	0.660	0.816
Carver County	0%	50%	0%	50%	0%	50%	0.632	0.667	0.681	0.588	0.704
Dakota County	0%	50%	0%	50%	0%	50%	0.533	0.601	0.669	0.668	0.811
Dukes County	0%	50%	0%	50%	0%	50%	0.471	0.522	0.576	0.581	0.748
Essex County	0%	25%	0%	25%	0%	25%	0.544	0.596	0.637	0.624	0.821
Franklin County	0%	50%	0%	50%	0%	50%	0.500	0.525	0.562	0.579	0.705
Hampden County	0%	50%	0%	25%	0%	25%	0.457	0.531	0.600	0.516	0.698
Hampshire County	0%	50%	0%	50%	0%	25%	0.500	0.519	0.581	0.552	0.702
Hennepin County	0%	50%	0%	50%	0%	50%	0.632	0.645	0.677	0.625	0.777
Hillsborough County	0%	50%	0%	50%	0%	50%	0.383	0.472	0.643	0.624	0.785
Manatee County	0%	50%	0%	50%	0%	50%	0.500	0.556	0.695	0.688	0.828
Mecklenburg County	0%	25%	0%	50%	0%	25%	0.534	0.619	0.659	0.615	0.753
Middlesex County	0%	50%	0%	25%	0%	25%	0.518	0.581	0.638	0.410	0.535
Milwaukee County	25%	50%	0%	25%	0%	25%	0.446	0.590	0.666	0.408	0.584
Monmouth County	0%	50%	0%	50%	0%	50%	0.519	0.625	0.698	0.507	0.609
Nantucket County	0%	50%	0%	50%	0%	50%	0.441	0.532	0.568	0.585	0.765
Norfolk County	0%	25%	0%	25%	0%	25%	0.554	0.601	0.644	0.642	0.824
New York City	0%	50%	0%	50%	0%	50%	0.637	0.752	0.810	0.797	0.929
Plymouth County	0%	50%	0%	50%	0%	50%	0.494	0.570	0.618	0.621	0.787
Ramsey County	0%	50%	0%	50%	0%	50%	0.558	0.636	0.664	0.636	0.810
Sarasota County	0%	50%	0%	50%	0%	50%	0.546	0.607	0.692	0.685	0.844
Suffolk County	0%	50%	0%	50%	0%	25%	0.591	0.688	0.743	0.731	0.906
Vanderburgh County	0%	50%	0%	25%	0%	25%	0.617	0.598	0.658	0.655	0.804
Washington County	25%	75%	0%	50%	0%	50%	0.556	0.634	0.659	0.604	0.725
Worcester County	0%	25%	0%	25%	0%	25%	0.482	0.543	0.596	0.346	0.473

**Table 2:** Approximate accuracy trends of the GHS-BUILT built-up surface datasets across different versions, gathered from evaluation studies based on the MTBF-33 reference dataset. Note that the reported F-1 scores are approximate averages across all counties, and that GHS-BUILT-R2018A was only evaluated in the state of Massachusetts, encompassing 14 out of 33 counties covered by MTBF-33.

GHSL release	Source data	Spatial resolution	Epoch	F1 (most rural)	F1 (most urban)	Data reference or related publication	Validation study
GHS_LDSMT_2015	Landsat	38 m	2014	0.11	0.85	https://doi.org/10.2788/253582	Leyk et al., 2018
GHS_LDSMT_2017	Landsat + Sentinel-1	30 m	2014	0.05	0.67	https://doi.org/10.1080/20964471.2019.1625528	Uhl et al., 2018
GHS-BUILT-S1	Sentinel-1	20 m	2016	0.05	0.60	https://doi.org/10.1080/20964471.2017.1397899	Uhl et al., 2018
GHS-BUILT-R2018A	Landsat + Sentinel-2	30 m	2014	0.45	0.80	https://doi.org/10.2905/jrc-ghsl-10007	Uhl & Leyk, 2022a
GHS-BUILT-S2	Sentinel-2	10 m	2018	0.52	0.63	https://doi.org/10.2905/016D1A34-B184-42DC-B586-E10B915DD863	This study

**Table 3:** Built-up quantity regression analysis results per county.

County	Slope	Intercept	R^2^	County	Slope	Intercept	R^2^
Anoka County	1.102	0.456	0.868	Manatee County	1.046	0.434	0.932
Baltimore County	1.020	−0.155	0.946	Mecklenburg County	0.549	1.913	0.965
Barnstable County	0.847	0.007	0.953	Middlesex County	0.873	−0.798	0.686
Berkshire County	0.602	0.064	0.928	Milwaukee County	0.654	1.462	0.958
Boulder County	0.655	−0.104	0.928	Monmouth County	0.994	0.939	0.697
Bristol County	0.936	−0.314	0.875	Nantucket County	0.904	0.412	0.930
Carver County	1.177	0.047	0.966	Norfolk County	0.853	−0.322	0.958
Dakota County	1.078	0.410	0.914	New York City	1.054	0.601	0.951
Dukes County	0.864	0.184	0.948	Plymouth County	0.823	0.065	0.900
Essex County	0.810	−0.335	0.964	Ramsey County	0.811	2.616	0.946
Franklin County	0.702	0.075	0.967	Sarasota County	1.018	0.689	0.918
Hampden County	0.441	0.395	0.958	Suffolk County	0.947	0.818	0.963
Hampshire County	0.570	0.161	0.858	Vanderburgh County	0.760	0.342	0.938
Hennepin County	0.983	1.470	0.908	Washington County	1.115	0.131	0.947
Hillsborough County	1.011	1.454	0.938	Worcester County	0.598	0.010	0.928

## Data Availability

The GHS-BUILT-S2 R2020A dataset is available at https://doi.org/10.2905/016D1A34-B184-42DC-B586-E10B915DD863. The MTBF-33 reference database is available at https://data.mendeley.com/datasets/w33vbvjtdy. Code for multi-resolution, global, zonal, and focal accuracy assessment is available at https://github.com/johannesuhl/local_accuracy.

## Footnotes

2 https://github.com/microsoft/GlobalMLBuildingFootprints.

3 https://github.com/microsoft/RoadDetections.

4 Our New York City study area encompasses the five boroughs of Bronx, Brooklyn, Manhattan, Queens, and Staten Island.

## Appendix

**Table A1:** Correlation coefficients between GHS built-up probability and built-up area fraction, as well as with focal accuracy / built-up surface density.

County	Pearson’s correlation coefficient of built-up probability and				
	BUFRAC	F1	Precision	Recall	Reference built-up density
Anoka County	0.284	0.086	0.042	0.084	0.521
Baltimore County	0.327	0.319	0.135	0.375	0.583
Barnstable County	0.407	0.168	0.114	0.119	0.464
Berkshire County	0.413	0.132	0.115	0.105	0.452
Boulder County	0.251	0.386	0.082	0.345	0.636
Bristol County	0.366	0.266	0.168	0.259	0.555
Carver County	0.290	0.047	−0.097	0.211	0.468
Dakota County	0.287	0.267	0.203	0.234	0.518
Dukes County	0.406	0.164	0.127	0.126	0.496
Essex County	0.342	0.230	0.123	0.218	0.562
Franklin County	0.408	0.137	0.120	0.089	0.425
Hampden County	0.394	0.110	0.239	0.020	0.476
Hampshire County	0.398	0.134	0.159	0.074	0.427
Hennepin County	0.223	0.194	0.188	0.120	0.535
Hillsborough County	0.285	0.340	0.354	0.245	0.529
Manatee County	0.335	0.327	0.272	0.316	0.548
Mecklenburg County	0.328	0.173	0.119	0.142	0.291
Middlesex County	0.339	0.311	0.171	0.306	0.594
Milwaukee County	0.141	0.358	0.241	0.312	0.389
Monmouth County	0.376	0.208	0.169	0.164	0.509
Nantucket County	0.372	0.226	0.155	0.163	0.543
Norfolk County	0.340	0.228	0.125	0.219	0.503
New York City	0.331	0.442	0.334	0.428	0.643
Plymouth County	0.384	0.204	0.132	0.187	0.482
Ramsey County	0.282	0.207	0.201	0.117	0.410
Sarasota County	0.322	0.260	0.229	0.240	0.503
Suffolk County	0.246	0.340	0.254	0.347	0.526
Vanderburgh County	0.370	0.256	0.164	0.204	0.555
Washington County	0.288	0.174	0.058	0.238	0.519
Worcester County	0.367	0.185	0.140	0.146	0.461

**Table A2:** Fitting polynomial functions to estimate built-up fraction from built-up probabilities, per county, and for polynomials of degree 1 to 4.

Polynomial degree	1st		2nd		3rd		4th	
County	RMSE	R^2^	RMSE	R^2^	RMSE	R2	RMSE	R^2^
Anoka County	29.136	0.081	28.588	0.115	28.307	0.132	28.268	0.135
Baltimore County	29.752	0.107	29.090	0.147	28.792	0.164	28.783	0.164
Barnstable County	24.492	0.166	24.140	0.190	24.072	0.194	24.050	0.196
Berkshire County	26.365	0.170	26.041	0.191	25.997	0.193	25.997	0.193
Boulder County	30.367	0.063	30.100	0.080	30.005	0.085	29.998	0.086
Bristol County	27.898	0.134	27.255	0.174	27.198	0.177	27.185	0.178
Carver County	29.310	0.084	28.729	0.120	28.407	0.140	28.368	0.142
Dakota County	30.341	0.082	29.908	0.108	29.570	0.128	29.544	0.130
Dukes County	23.454	0.165	23.168	0.185	23.112	0.189	23.100	0.190
Essex County	27.865	0.117	27.593	0.134	27.557	0.136	27.545	0.137
Franklin County	25.984	0.167	25.611	0.190	25.561	0.193	25.559	0.194
Hampden County	27.593	0.155	27.183	0.180	27.116	0.184	27.114	0.184
Hampshire County	26.714	0.158	26.397	0.178	26.339	0.182	26.337	0.182
Hennepin County	30.948	0.050	30.582	0.072	30.438	0.081	30.421	0.082
Hillsborough County	29.727	0.081	29.508	0.095	29.497	0.095	29.483	0.096
Manatee County	29.343	0.112	29.195	0.121	29.182	0.122	29.164	0.123
Mecklenburg County	30.514	0.108	30.038	0.135	29.791	0.150	29.779	0.150
Middlesex County	28.514	0.115	28.205	0.134	28.181	0.135	28.173	0.136
Milwaukee County	31.189	0.020	30.589	0.057	30.580	0.058	30.501	0.063
Monmouth County	27.446	0.142	26.481	0.201	26.375	0.207	26.327	0.210
Nantucket County	24.205	0.138	23.958	0.156	23.930	0.158	23.927	0.158
Norfolk County	28.612	0.116	28.183	0.142	28.139	0.145	28.122	0.146
New York City	30.049	0.110	29.095	0.165	29.015	0.170	28.948	0.174
Plymouth County	26.442	0.148	25.938	0.180	25.863	0.185	25.844	0.186
Ramsey County	29.582	0.080	29.317	0.096	29.204	0.103	29.184	0.104
Sarasota County	29.233	0.104	29.107	0.112	29.088	0.113	29.077	0.113
Suffolk County	30.991	0.061	30.506	0.090	30.493	0.091	30.466	0.092
Vanderburgh County	28.985	0.137	28.545	0.163	28.471	0.167	28.460	0.168
Washington County	29.124	0.083	28.563	0.118	28.254	0.137	28.213	0.139
Worcester County	27.916	0.135	27.609	0.154	27.578	0.156	27.573	0.156

**Table A3:** Sensitivity of overall accuracy metrics to the analytical unit (i.e., grid cell block sizes).

County	Precision (1×1)	Precision (3×3)	Precision (5×5)	Recall (1×1)	Recall (3×3)	Recall (5×5)	F1 (1×1)	F1 (3×3)	F1 (5×5)
Anoka County	0,612	0,764	0,841	0,554	0,673	0,727	0,581	0,715	0,780
Baltimore County	0,672	0,816	0,858	0,669	0,792	0,842	0,671	0,804	0,850
Barnstable County	0,666	0,835	0,898	0,567	0,775	0,872	0,613	0,803	0,884
Berkshire County	0,731	0,845	0,882	0,461	0,606	0,692	0,565	0,706	0,776
Boulder County	0,683	0,819	0,862	0,320	0,438	0,478	0,436	0,571	0,615
Bristol County	0,700	0,844	0,889	0,624	0,790	0,860	0,660	0,816	0,874
Carver County	0,610	0,758	0,820	0,568	0,657	0,693	0,588	0,704	0,752
Dakota County	0,626	0,769	0,830	0,716	0,858	0,909	0,668	0,811	0,867
Dukes County	0,604	0,746	0,807	0,560	0,749	0,826	0,581	0,748	0,817
Essex County	0,714	0,879	0,920	0,554	0,770	0,857	0,624	0,821	0,887
Franklin County	0,676	0,772	0,810	0,506	0,648	0,729	0,579	0,705	0,768
Hampden County	0,747	0,868	0,912	0,394	0,583	0,728	0,516	0,698	0,809
Hampshire County	0,719	0,832	0,872	0,447	0,607	0,711	0,552	0,702	0,784
Hennepin County	0,645	0,795	0,862	0,606	0,759	0,805	0,625	0,777	0,832
Hillsborough County	0,583	0,735	0,778	0,671	0,842	0,907	0,624	0,785	0,838
Manatee County	0,658	0,799	0,833	0,721	0,858	0,900	0,688	0,828	0,865
Mecklenburg County	0,763	0,873	0,916	0,516	0,663	0,778	0,615	0,753	0,841
Middlesex County	0,717	0,870	0,914	0,287	0,386	0,430	0,410	0,535	0,585
Milwaukee County	0,697	0,881	0,925	0,289	0,437	0,483	0,408	0,584	0,635
Monmouth County	0,651	0,792	0,848	0,414	0,495	0,528	0,507	0,609	0,651
Nantucket County	0,585	0,733	0,795	0,586	0,800	0,874	0,585	0,765	0,832
Norfolk County	0,712	0,868	0,915	0,585	0,784	0,875	0,642	0,824	0,895
New York City	0,771	0,916	0,929	0,824	0,944	0,975	0,797	0,929	0,951
Plymouth County	0,681	0,827	0,881	0,571	0,750	0,837	0,621	0,787	0,859
Ramsey County	0,682	0,828	0,886	0,596	0,793	0,859	0,636	0,810	0,872
Sarasota County	0,663	0,823	0,872	0,710	0,865	0,922	0,685	0,844	0,896
Suffolk County	0,742	0,899	0,914	0,720	0,914	0,955	0,731	0,906	0,934
Vanderburgh County	0,738	0,862	0,901	0,589	0,754	0,832	0,655	0,804	0,865
Washington County	0,621	0,762	0,823	0,588	0,691	0,734	0,604	0,725	0,776
Worcester County	0,727	0,854	0,896	0,227	0,327	0,385	0,346	0,473	0,538

**Table A4:** Regression analysis of focal reference and GHSL-based built-up quantity per county and support level.

Spatial support	1 km			2.5 km			5 km		
County	Slope	Intercept	R^2^	Slope	Intercept	R^2^	Slope	Intercept	R^2^
All counties	0.874	0.203	0.868	0.878	0.108	0.883	0.886	0.033	0.888
Anoka County	1.102	0.456	0.946	1.111	0.390	0.971	1.122	0.321	0.980
Baltimore County	1.020	−0.155	0.953	1.050	−0.369	0.979	1.061	−0.426	0.990
Barnstable County	0.847	0.007	0.928	0.864	−0.078	0.951	0.888	−0.217	0.959
Berkshire County	0.602	0.064	0.928	0.614	0.026	0.971	0.629	0.004	0.985
Boulder County	0.655	−0.104	0.875	0.585	0.059	0.821	0.481	0.151	0.742
Bristol County	0.936	−0.314	0.966	0.954	−0.400	0.977	0.961	−0.420	0.981
Carver County	1.177	0.047	0.914	1.220	−0.058	0.962	1.251	−0.116	0.984
Dakota County	1.078	0.410	0.948	1.101	0.258	0.976	1.116	0.179	0.985
Dukes County	0.864	0.184	0.964	0.878	0.130	0.983	0.889	0.095	0.991
Essex County	0.810	−0.335	0.967	0.821	−0.397	0.981	0.826	−0.418	0.987
Franklin County	0.702	0.075	0.958	0.726	0.029	0.978	0.753	−0.001	0.983
Hampden County	0.441	0.395	0.858	0.435	0.515	0.863	0.445	0.440	0.902
Hampshire County	0.570	0.161	0.908	0.590	0.088	0.950	0.611	0.031	0.964
Hennepin County	0.983	1.470	0.938	0.990	1.349	0.957	0.991	1.258	0.972
Hillsborough County	1.011	1.454	0.932	1.037	0.991	0.968	1.047	0.809	0.980
Manatee County	1.046	0.434	0.965	1.048	0.236	0.985	1.051	0.187	0.991
Mecklenburg County	0.549	1.913	0.686	0.520	2.281	0.746	0.490	2.608	0.792
Middlesex County	0.873	−0.798	0.958	0.891	−0.938	0.971	0.887	−0.896	0.978
Milwaukee County	0.654	1.462	0.697	0.693	0.184	0.785	0.715	−0.518	0.856
Monmouth County	0.994	0.939	0.930	1.033	0.565	0.967	1.045	0.413	0.982
Nantucket County	0.904	0.412	0.958	0.936	0.196	0.980	0.964	0.102	0.989
Norfolk County	0.853	−0.322	0.951	0.877	−0.539	0.968	0.914	−0.846	0.976
New York City	1.054	0.601	0.900	1.071	0.008	0.914	1.099	−0.876	0.929
Plymouth County	0.823	0.065	0.946	0.831	0.017	0.973	0.831	0.001	0.982
Ramsey County	0.811	2.616	0.918	0.812	2.460	0.948	0.829	1.992	0.963
Sarasota County	1.018	0.689	0.963	1.023	0.484	0.986	1.030	0.343	0.994
Suffolk County	0.947	0.818	0.938	0.946	0.762	0.950	0.946	0.690	0.955
Vanderburgh County	0.760	0.342	0.947	0.756	0.336	0.969	0.756	0.306	0.979
Washington County	1.115	0.131	0.928	1.140	0.041	0.957	1.177	−0.081	0.971
Worcester County	0.598	0.010	0.900	0.601	−0.057	0.917	0.616	−0.189	0.914
